# Changes of physico-chemical properties of nano-biomaterials by digestion fluids affect the physiological properties of epithelial intestinal cells and barrier models

**DOI:** 10.1186/s12989-022-00491-w

**Published:** 2022-07-19

**Authors:** Giulia Antonello, Arianna Marucco, Elena Gazzano, Panagiotis Kainourgios, Costanza Ravagli, Ana Gonzalez-Paredes, Simone Sprio, Esperanza Padín-González, Mahmoud G. Soliman, David Beal, Francesco Barbero, Paolo Gasco, Giovanni Baldi, Marie Carriere, Marco P. Monopoli, Costas A. Charitidis, Enrico Bergamaschi, Ivana Fenoglio, Chiara Riganti

**Affiliations:** 1 Department of Chemistry, University of Turin, Via Pietro Giuria 7, 10125 Turin, Italy; 2 Department of Public Health and Pediatrics, University of Turin, Piazza Polonia, 94, 10126 Turin, Italy; 3 Department of Oncology, University of Turin, Via Santena 5 bis, 10126 Turin, Italy; 4 Department of Life Sciences and Systems Biology, University of Turin, Via Accademia Albertina 13, 10123 Turin, Italy; 5 Research Unit of Advanced, Composite, Nano-Materials and Nanotechnology, School of Chemical Engineering, National Technical University of Athens, 9 Heroon Polytechniou St., 15780 Zographos, Athens, Greece; 6 Colorobbia Consulting Srl, Headwork, Via Pietramarina, 53, 50059 Sovigliana, Vinci, FI Italy; 7 Nanovector Srl, Headwork, Via Livorno 60, 10144 Turin, Italy; 8 National Research Council, Institute of Science and Technology for Ceramics ISTEC-CNR, Via Granarolo 64, 48018 Faenza, RA Italy; 9 Department of Chemistry, Royal College of Surgeons in Ireland (RCSI), 123 St Stephen Green, Dublin 2, Ireland; 10 CEA, CNRS, IRIG, SyMMES-CIBEST, Université Grenoble Alpes, 38000 Grenoble, France

**Keywords:** Nano-biomaterials, In vitro simulated digestion, Biotransformation, Toxicity, Caco-2, HCT116, HCoEpiC, Gastro-intestinal barrier, Permeability, Inflammation

## Abstract

**Background:**

The widespread use of nano-biomaterials (NBMs) has increased the chance of human exposure. Although ingestion is one of the major routes of exposure to NBMs, it is not thoroughly studied to date. NBMs are expected to be dramatically modified following the transit into the oral-gastric-intestinal (OGI) tract. How these transformations affect their interaction with intestinal cells is still poorly understood. NBMs of different chemical nature—lipid-surfactant nanoparticles (LSNPs), carbon nanoparticles (CNPs), surface modified Fe_3_O_4_ nanoparticles (FNPs) and hydroxyapatite nanoparticles (HNPs)—were treated in a simulated human digestive system (SHDS) and then characterised. The biological effects of SHDS-treated and untreated NBMs were evaluated on primary (HCoEpiC) and immortalised (Caco-2, HCT116) epithelial intestinal cells and on an intestinal barrier model.

**Results:**

The application of the in vitro SDHS modified the biocompatibility of NBMs on gastrointestinal cells. The differences between SHDS-treated and untreated NBMs could be attributed to the irreversible modification of the NBMs in the SHDS. Aggregation was detected for all NBMs regardless of their chemical nature, while pH- or enzyme-mediated partial degradation was detected for hydroxyapatite or polymer-coated iron oxide nanoparticles and lipid nanoparticles, respectively. The formation of a bio-corona, which contains proteases, was also demonstrated on all the analysed NBMs. In viability assays, undifferentiated primary cells were more sensitive than immortalised cells to digested NBMs, but neither pristine nor treated NBMs affected the intestinal barrier viability and permeability. SHDS-treated NBMs up-regulated the tight junction genes (claudin 3 and 5, occludin, zonula occludens 1) in intestinal barrier, with different patterns between each NBM, and increase the expression of both pro- and anti-inflammatory cytokines (IL-1β, TNF-α, IL-22, IL-10). Notably, none of these NBMs showed any significant genotoxic effect.

**Conclusions:**

Overall, the results add a piece of evidence on the importance of applying validated in vitro SHDS models for the assessment of NBM intestinal toxicity/biocompatibility. We propose the association of chemical and microscopic characterization, SHDS and in vitro tests on both immortalised and primary cells as a robust screening pipeline useful to monitor the changes in the physico-chemical properties of ingested NBMs and their effects on intestinal cells.

**Supplementary Information:**

The online version contains supplementary material available at 10.1186/s12989-022-00491-w.

## Background

In the last few years, nano-biomaterials (NBMs) have been widely used for manufacturing innovative food packaging [1, 2] nutraceuticals [3], cosmetics [4, 5], as well as in dentistry [6], precision medicine [7–9] and agriculture [10, 11], increasing the likelihood of human exposure through ingestion and transit through the gastro-intestinal (GI) tract [12].

A growing number of studies suggested a possible interference of ingested NBMs with the gut microenvironment [13]. The human digestive apparatus is composed of many sections with different structures and functions. The most complex part is the intestinal tract. In particular, the small intestine mediates the absorption of nutrients through transcellular processes or paracellular diffusion. The latter is limited by the presence of tight junction (TJs) complexes, formed by zonula occludens-1, occludin and claudin proteins [14]. In physiological conditions, TJs prevent water and electrolyte leakage and avoid lumen infections. However, some conditions, such as inflammatory bowel disease [15, 16], can alter the structure of TJs, increasing the intestinal barrier permeability. Two studies report that nanometric SiO_2_ or TiO_2_ can induce similar effects [17, 18].

While a substantial amount of knowledge has been accumulated for the inhalation route, only recently the fate of the ingested NBMs has gained interest in the nanotoxicology community [19]. The poor awareness of ingestion’s relevance as exposure route to NBMs, the non-suitability of the available models, and the lack of consensus on the most suitable in vitro models reproducing the complexity of the Oral-Gastro-Intestinal (OGI) tract are the main reasons for this delay.

In the last few years, some models to mimic the intestinal barrier in vitro and study the toxicity of NBM have been proposed, as co-cultures of different intestinal cell types. Co-culture monolayers, composed by enterocyte-like cells with TJs and brush border (Caco-2), and goblet cells secreting mucus (HT29-MTX), have been used to investigate the toxicity of NBMs such as TiO_2_ nanoparticles [20–23] and multi walled carbon nanotubes [24]. The effect of halloysite clay nanotube on intestinal barrier [25] has been also studied on Caco-2/HT29-MTX co-culture plus Raji B cells (that promote Caco-2 differentiation into M cells, characterised by the typical digestive function of enterocytes). The results have indicated the absence of cytotoxicity despite the high production of pro-inflammatory cytokines and the increase in cell growth and proliferation [26].

Recently, newly intestinal mucosa 3D models have been developed culturing Caco-2 cells on a layer of macrophages and dendritic cells embedded in collagen scaffolds. Indeed, these immune cells are both present in the intestinal lamina propria and react to inflammatory stimuli, by producing pro-inflammatory cytokines (e.g. interleukin (IL)-6, tumour necrosis factor (TNF)-α) and anti-inflammatory mediator (e.g. IL-10) [27, 28], as a possible compensation mechanism. Interestingly, some of these mediators such as IL-6 and TNF-α are involved in the pathogenesis of inflammatory bowel disease, promoting gut damage and loss of intestinal barrier integrity, while IL-10 reduces the inflammation typically associated with this pathology [29]. Some NBMs have been shown to induce intestinal cells to assume the phenotype of inflammatory bowel disease. For instance, the model proposed by Susewind and co-workers to assess the safety of TiO_2_, Ag and Au nanoparticles [30] is characterised by the loss of barrier function and the increased production of inflammatory cytokines that regress after the treatment with anti-inflammatory drugs [31], well recapitulating the situation occurring in vivo.

Furthermore, some authors proposed *ex-vivo* systems derived from murine, porcine, or human bowel to study the intestinal permeability after the exposure to nanoparticles [32, 33].

Although these models are closer to the intestinal anatomy and physiology than conventional monocultures, the most used model for the evaluation of the exposure to NBMs is still the culture of Caco-2 cells on porous membrane inserts. In these conditions the cells rapidly differentiate into an intestinal barrier [34–37]. In addition the HCT116 model, another colon cancer cell line, is a widely accepted tool to evaluate the genotoxicity of NBMs [38–41].

Another important issue to be considered in in vitro intestinal models is the biological identity of the NBMs that are in contact with cells, because ingested NBMs interact with different fluids characterised by specific pH, ionic strength, and composition. This interaction may dramatically modify NBMs’ properties. For instance, the exposure may lead to dissolution [42], aggregation/agglomeration [43], and formation of bio-molecular corona [44] that may change over time. Monitoring such biotransformations is crucial to understand the NBMs biological fate in the gut environment and the impact on their toxicity [19, 45].

Recently, several in vitro digestion models have been proposed to investigate the digestion-driven modifications of NBMs. Sequential incubations in simulated gastric and intestinal fluids have been used to study the bioactivity of starch nanocapsules [46] and zein-pectin nanoparticles [47]. A similar protocol, improved with longer incubation times and the addition of simulated saliva, has been set up to study the dissolution of Fe_2_O_3_ nanoparticles [48], the agglomeration of TiO_2_ nanoparticles and their interaction with proteins [49]. Several other models have been proposed, differing in fluid composition or incubation times, such as the simulated digestion system reported in Sohal et al. [50] and slightly modified by Marucco et al. [39]. Most of these models have been used to describe the transformation occurring to NBMs during the digestive process. Nevertheless, few studies have been published on the effects that such changes have on intestinal cells [51–54].

To fill this gap, in this study we adopted a simulated human digestion system (SHDS) consisting of sequential incubations in simulated saliva fluid (SSF), simulated gastric fluid (SGF) and simulated intestinal fluid (SIF) to investigate the biotransformation of NBMs of different chemical nature. Samples representative of NBMs with potential applications in oral drug delivery or nutraceutical field have been selected, i.e. hydroxyapatite, carbon nanoparticles, lipid-surfactant nanoparticles and surface modified magnetite nanoparticles [55–58]. The effects of SHDS-treated or untreated NBMs on viability, barrier integrity and intracellular inflammation were evaluated on primary and immortalised epithelial intestinal cells.

## Results

### Properties of the NBMs

In this study, we selected the following NBMs: three colloidal formulations composed by elemental carbon nanoparticles (CNPs), lipid-surfactant nanoparticles (LSNPs) and PLGA-PEG coated magnetite nanoparticles (FNPs), and one powder sample of hydroxyapatite nanoparticles (HNPs).

The main properties of the materials are summarized in Table 1.

**Table 1:** Physico-chemical properties of samples

Samples	Appearance	Concentration (mg/ml)	Z-average hydrodynamic diameter (nm)*	ζ-potential (mV)**	Suspension pH**
LSNPs Lipid-surfactant nanoparticles	Colloidal suspension	12	135.0 ± 0.5 PDI 0.244	−16.3 ± 1.5	5.46
CNPs Carbon nanoparticles	Colloidal suspension	1.2	130.8 ± 1.0 PDI 0.170	−52.6 ± 1.0	4.60
FNPs PLGA-PEG coated Fe_3_O_4_ nanoparticles	Colloidal suspension	2	83.2 ± 0.5 PDI 0.169	−40.8 ± 1.0	6.84
HNPs Hydroxyapatite nanoparticles	Powder	1	3126 ± 523 PDI 0.648	−2.0 ± 0.2	7.75

PDI is referred to Polydispersity Index

^*^DLS measurement, samples diluted in water (100 µg/ml)

^**^ELS and pH measurement, samples diluted in water (100 µg/ml)

**Fig. 1:**
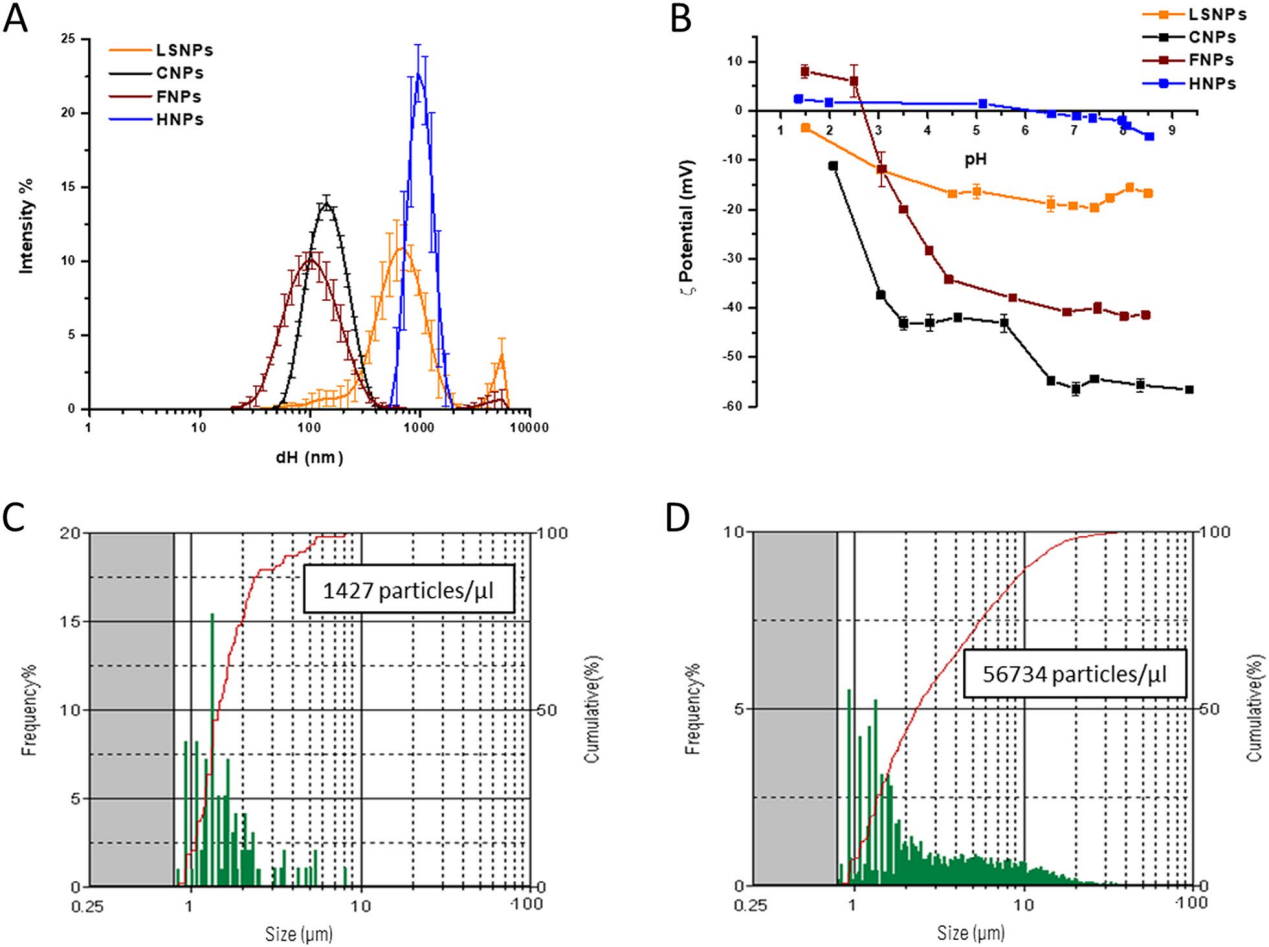
Size distribution and ζ-potential of the NBMs. **A** Hydrodynamic diameter (d_H_) distribution of the samples in water (100 µg/ml), evaluated by DLS. Each line represents the mean values of 15 measurements that were obtained in three independent experiments. ± SD; **B** ζ-potential versus pH of the samples evaluated by ELS; **C**, **D** Size distribution of **C** LSNPs and **D** HNPs evaluated by FPIA. The concentration is reported as the number of micrometric particles

The size distribution and the surface properties of the NBMs were investigated by Dynamic Light Scattering (DLS) and Electrophoretic Light Scattering (ELS), respectively (Table 1 and Fig. 1A and B).

Because of the presence of particles/aggregates larger than the upper limit of detection of the DLS technique (5 µm), LSNPs and HNPs were also analysed by flow particle imaging analysis (FPIA) (size range 1–150 µm) (Fig. 1C and D).

Based on the low polydispersity index (PDI) values and on the small standard deviation (SD) of the size distribution (Table 1), CNPs and FNPs appeared stable and monodisperse colloidal suspensions. CNPs and FNPs were mainly composed of nanometric particles (< 100 nm), albeit particles/agglomerates or aggregates in the nanometric/sub-micrometric range were detected as well (Fig. 1A). According to DLS, LSNPs had a larger size than CNPs and FNPs, mainly in the sub-micrometric range (Fig. 1A). However, it might correspond to the presence of few sub-micrometric particles, since DLS techniques overestimate the abundance of particles with larger sizes. FPIA confirms the presence of few particles with a diameter between 1 and 10 μm (Fig. 1C). As far as HNPs are concerned, particles were distributed in a wide range of sizes, from 400 nm to 20 μm (Fig. 1D). In water HNPs formed unstable suspensions, with clear sedimentation during time. More details on the structure of the four NBMs were provided by transmission electron microscope (TEM) analysis (following section). As expected, LSNPs and CNPs exhibited negative ζ-potential values in the whole pH range (Fig. 1B), suggesting the presence of negatively charged surface groups. FNPs showed positive ζ-potential values only at very low pH values (Fig. 1B), likely because of the contribution of the magnetite core. HNPs had ζ-potential values close to 0 mV at all pH (Fig. 1B) that well agree with the instability of the suspensions.

### Effect of the simulated human digestive system (SHDS) on the measured particles size

The transformation of the NBMs was monitored in terms of changes in size distribution, surface modifications, and degradation or dissolution by enzymatic digestion.

**Fig. 2:**
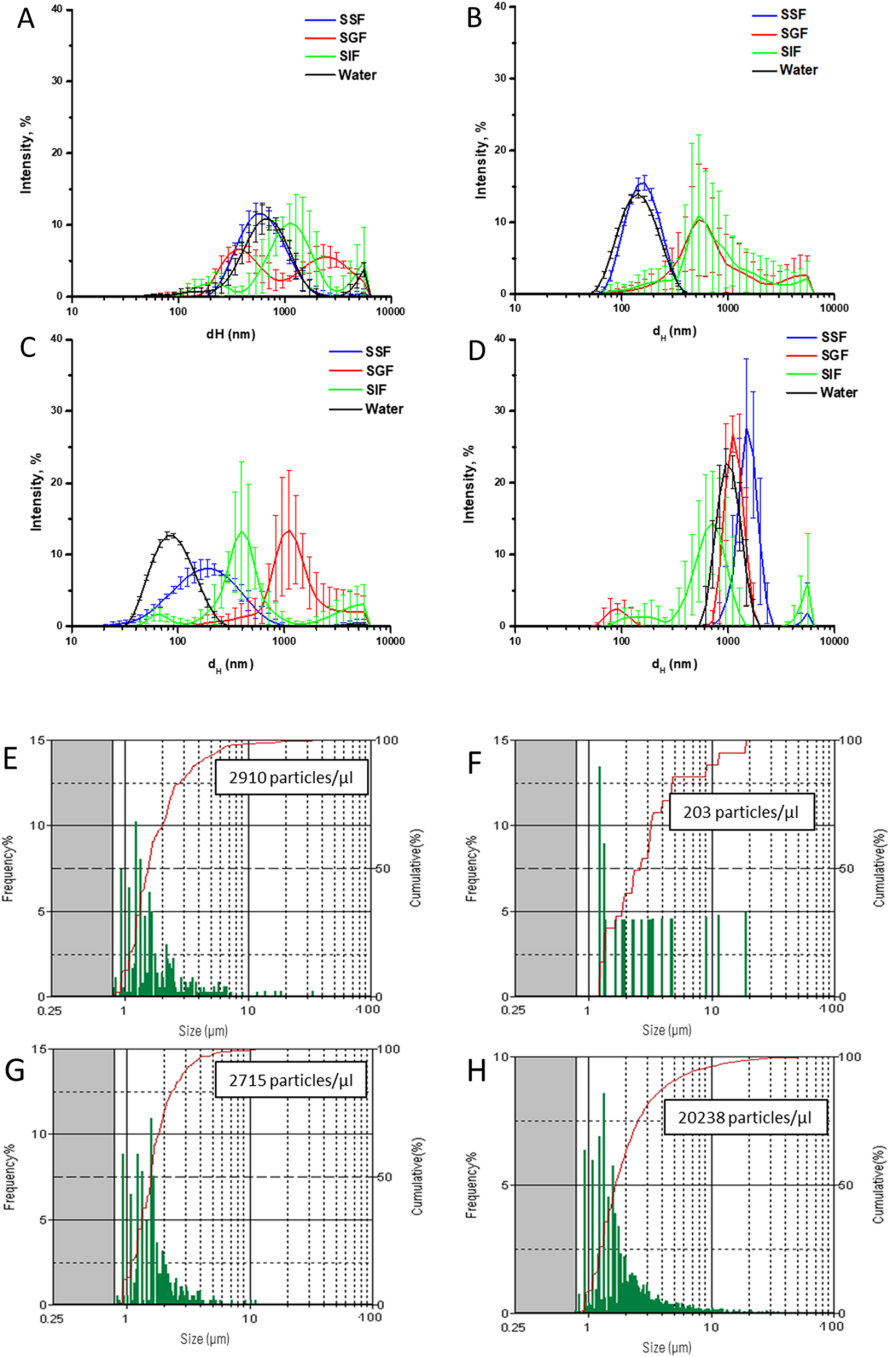
Size distribution changes after incubation with the SHDS. Upper panels: DLS patterns of the NBMs in the different fluids of the SHDS; **A** LSNPs; **B** CNPs, **C** FNPs; **D** HNPs. Each line represents the mean values of 15 measurements that were obtained in three independent experiments. Lower panels: FPIA size distribution of the NBMs after SHDS; **E** LSNPs; **F** CNPs, **G** FNPs; **H** HNPs. The concentration is reported as the number of micrometric particles. Each line is the mean of three independent experiments ± SD

The changes in size distribution of NBMs during the SHDS treatment were firstly measured by integrating DLS and FPIA data. In Fig. 2 the hydrodynamic distribution in the different compartments was compared with those measured in water.

No changes of size distribution were found for LSNPs and CNPs in SSF (Fig. 2A and B), while a shift of the distribution curve toward higher diameters was observed for FNPs (Fig. 2C), suggesting agglomeration/aggregation. In the SGF a clear destabilization of the colloidal suspensions was observed for all NBMs, as inferred by the diameters shift towards high values and the increase in the standard deviation among the measurements (Fig. 2A–D). This was expected, because of the low pH and high ionic strength of the media. Because of the intrinsic instability of HNPs suspension, the effect of the SGF was less evident (Fig. 2D). The suspensions remained highly unstable also in the SIF and the presence of large aggregates was optically visible in the final suspension (Additional file 1: Fig S1). The presence of micrometric particles/aggregates or agglomerates was evaluated by FPIA (Fig. 2E–H). Micrometric particles were detected in all cases, albeit in different amounts. LSNPs (Fig. 2E) exhibited a concentration of micrometric particles higher than the untreated material, while for HNPs (Fig. 2H) a decrease was observed.

The size distribution was also measured by incubating the NBMs directly in the intestinal fluid (Additional file 1: Fig S2). In this case, the suspension appeared more stable and less aggregated than after the SHDS, suggesting that the aggregation occurred in the SGF, and was irreversible for all NBMs.

**Fig. 3:**
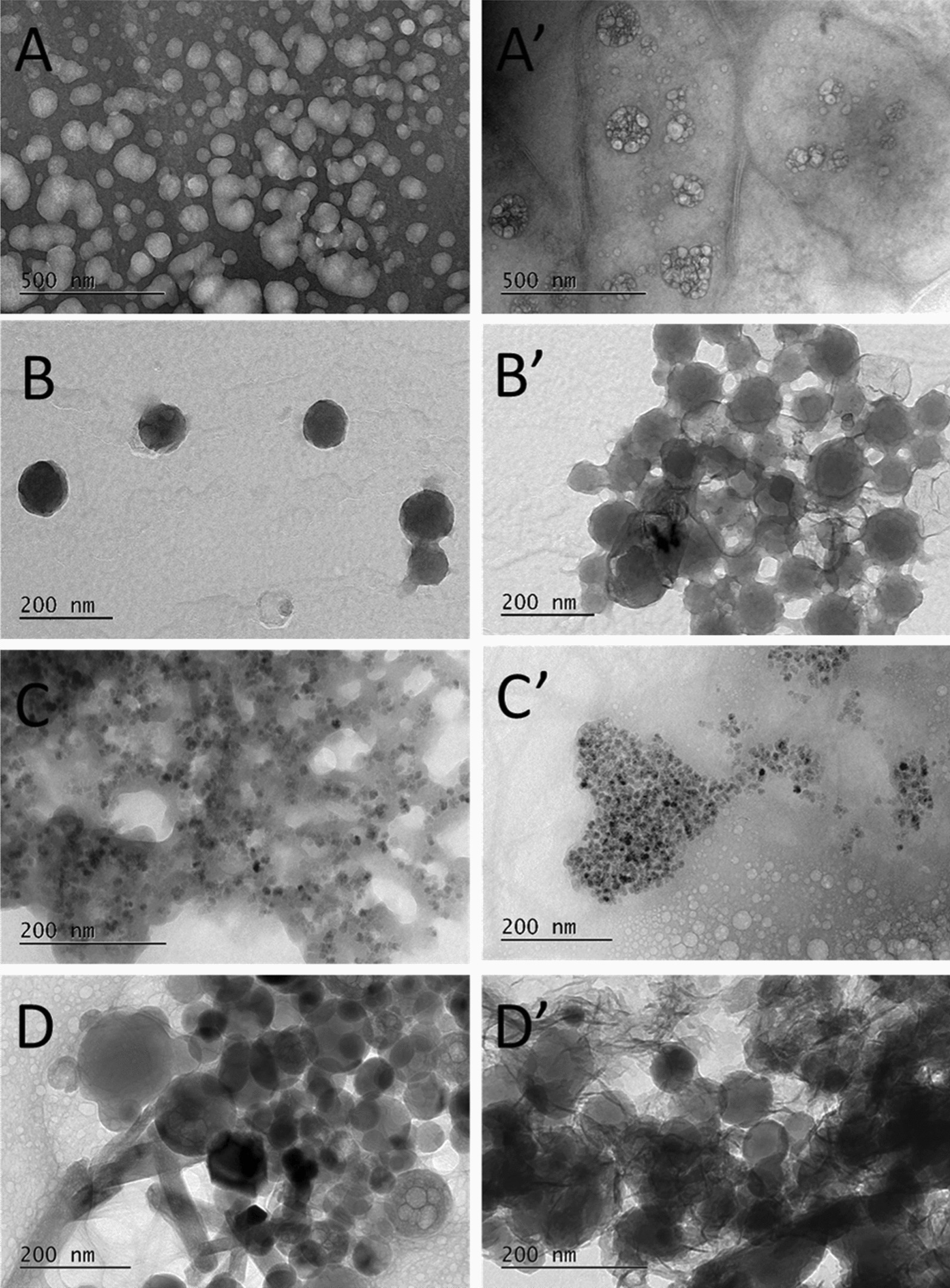
Transformation of NBMs monitored by TEM analysis. Representative TEM images of **A** LSNPs; **B** CNPs, **C** FNPs; **D** HNPs before SHDS-treatment and **A**′ LSNPs; **B**′ CNPs, **C**′ FNPs; **D**′ HNPs after SHDS-treatment

TEM analysis of the SHDS-treated and untreated NBMs was also performed (Fig. 3).

Untreated LSNPs (Fig. 3A) were composed by quasi-spherical particles of different size, confirming the DLS analysis (Fig. 1A). SHDS-treated LSNPs (Fig. 3A′) appeared of smaller dimensions, suggesting degradation, but organized in large aggregates surrounded by biological material deriving from the SHDS fluids.

Untreated CNPs (Fig. 3B) appeared spherical, well dispersed, and had a narrow size distribution around 120 nm, in agreement with the DLS data, whereas the SHDS-treated CNPs (Fig. 3B′) resulted in an agglomerated and entangled state with associated biological material, similarly to LSNPs (Fig. 3A′).

Untreated FNPs (Fig. 3C) appeared as small spherical iron oxide particles embedded inside the PLGA-PEG polymer matrix. After the SHDS (Fig. 3C′) the polymer matrix was apparently removed, likely because of the biodegradable nature of the PLGA polymer [59]. Transparent spherical structures, which can be attributed to the polymeric residues still present after a partial biodegradation or to the biological matrix, were visible. Iron oxide particles appeared highly aggregated, because of the degradation of the polymeric matrix, in line with the results obtained by the DLS analyses (Fig. 2C). No significant alterations of the iron oxide particles morphology were observed.

Large particles of very different shapes such as rods, rectangles or spheres were observed for HNPs (Fig. 3D). This shape/size diversity can justify the instability of the colloidal suspensions and the inconclusive results in the DLS analysis. Nevertheless, SHDS-treated HNPs (Fig. 3D′) apparently underwent a dramatic transformation in terms of morphology with evident biological material surrounding the HNPs. Being soluble at acidic pH (Additional file 1: Fig S3) HNPs are expected to dissolve in the SGF [60], and eventually re-precipitate in the SIF.

On the other hand, LSNPs were likely subjected to hydrolysis by lipases [61]. To investigate the susceptibility of LSNPs to enzymatic degradation, this NBM was incubated in a solution of lipase in water at the same pH as the intestinal fluid, and the size distribution was monitored up to 24 h (Additional file 1: Fig S4). A shift of the d_H_ distribution towards lower values was observed already after 15 min (Additional file 1: Fig S4A) indicating degradation; a decrease of approximately 8% in the mean d_H_ value occurred after 24 h (Additional file 1: Fig S4B). These data confirm the partial degradation of LSNPs observed by TEM analysis (Fig. 3A′).

### Bio-molecular corona formation during SHDS

The formation of a bio-molecular corona was investigated on CNPs and FNPs, because these NBMs exhibit a surface reactivity that can be used as a probe to monitor the extent of coverage of the surface.

**Fig. 4:**
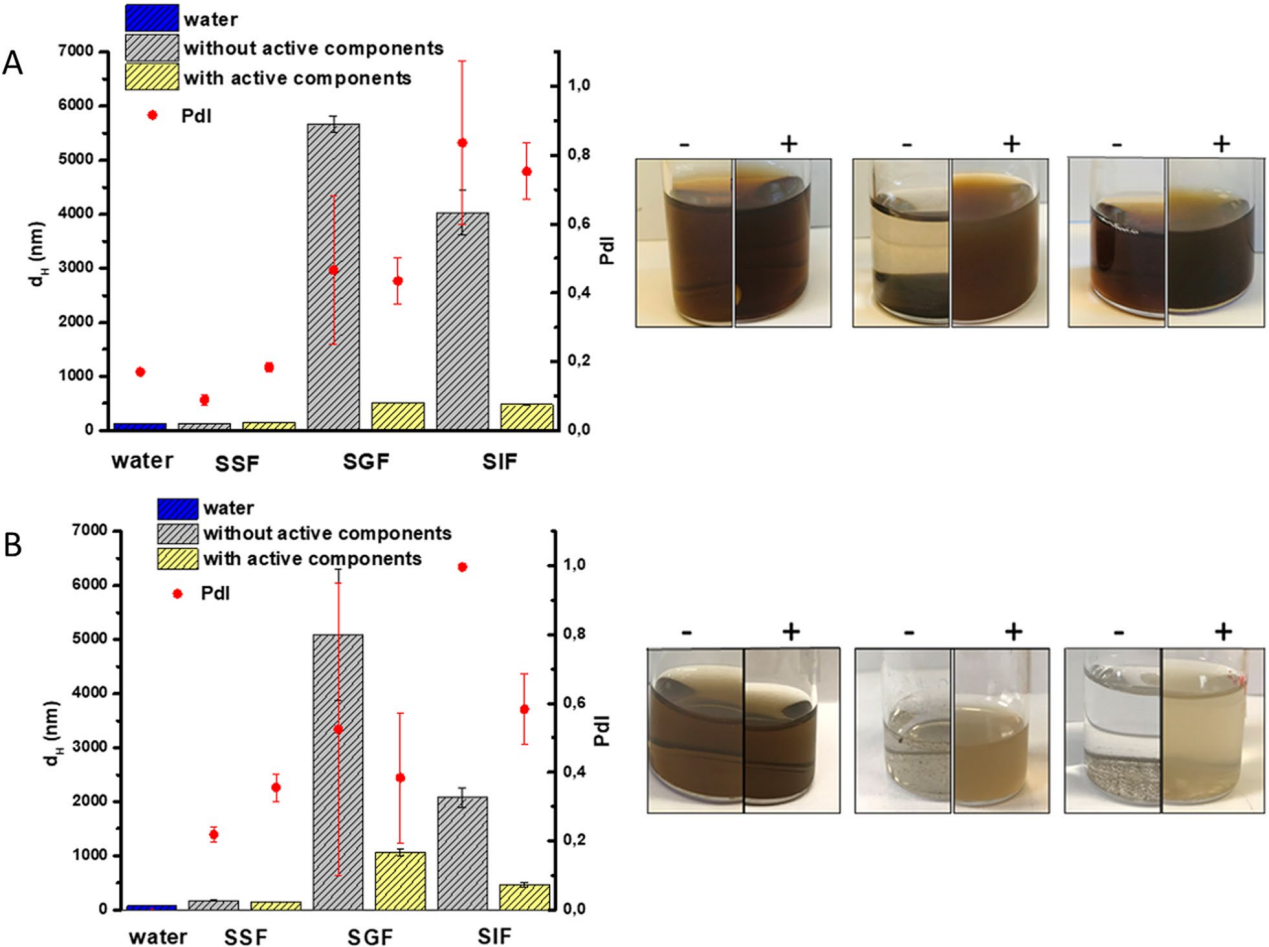
Effect of the active components on NBMs aggregation. Mean d_H_ and PDI (left panels) and representative images of the suspensions (right panels) of **A** CNPs and **B** FNPs in the various OGI compartments in the presence or absence of active components. Each line represents the mean values of 15 measurements that were obtained in three independent experiments

The SHDS was performed firstly with and without active components (proteins, bile and uric acid, Table 4), and the size distribution changes were monitored (Fig. 4).

In both SGF and SIF a higher Z-average and PDI values in the absence of active components was observed compared to fluids with active components. The suspensions were largely unstable, and the particles slowly deposited on the bottom of the flask, as shown in Fig. 4. This confirms the formation of a bio-molecular corona in the SHDS that stabilizes the colloids.

**Fig. 5:**
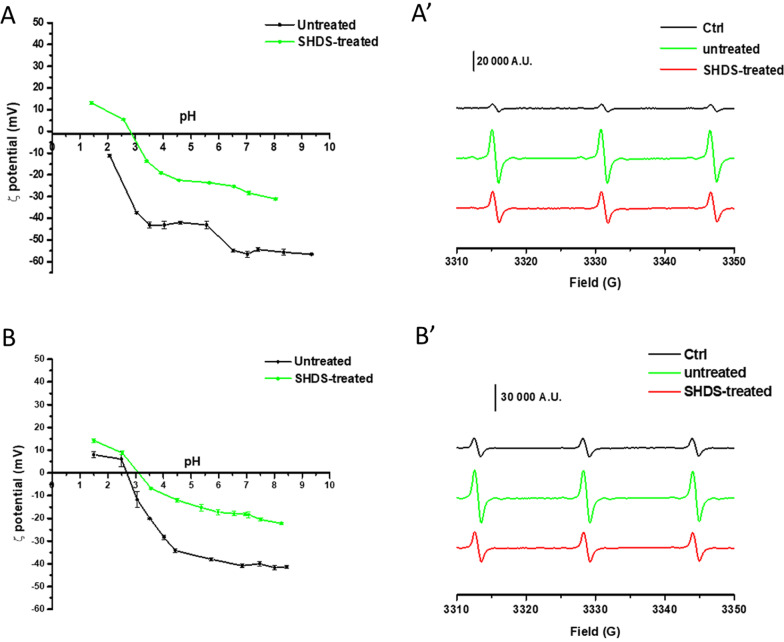
Extent of surface coverage and reversibility of the bio-molecular corona. Left panels: ζ-potential versus pH of **A** CNPs; **B** FNPs treated with SHDS and washed to remove the soft corona in comparison with the untreated one; right panels: EPR spectra recorded on **A**′ CNPs; **B**′ FNPs treated with the SHDS in comparison with the untreated one, using the spin-probe TEMPONE-H (Ctrl = no particles)

To gain information on the extent of the surface coverage, the NBMs were analysed for their surface charge by ELS and reactivity by electron paramagnetic resonance (EPR) spectroscopy (Fig. 5). The experiments were performed after washing steps aimed at removing the soft corona.

The ζ-potential was measured in ultrapure water by varying the pH of the suspension (Fig. 5A and B). The ζ-potential curves of CNPs treated with SHDS were different compared to untreated CNPs, suggesting the presence of biomolecules at the surface. Similarly, treated FNPs showed a ζ-potential shift, which might be due to the presence of a bio-molecular corona, and/or to the removal of the polymeric coating.

The surface reactivity was monitored by using the spin-probe TEMPONE-H. This is an unspecific probe able to react with Reactive Oxygen Species with redox-active surface centres leading to the stable radical TEMPONE, detectable by EPR spectroscopy [62]. Therefore, this system is suitable to monitor the surface reactivity of nanomaterials.

In the presence of untreated CNPs (Fig. 5A′) or FNPs (Fig. 5B′), the typical three-line signal of the TEMPONE radicals was observed. When treated with SHDS the surface reactivity of both FNPs and CNPs decreased, but was not eliminated, suggesting that the surface was still partially exposed to the solvent.

### NBMs identity in cell culture medium and in SHDS

**Fig. 6:**
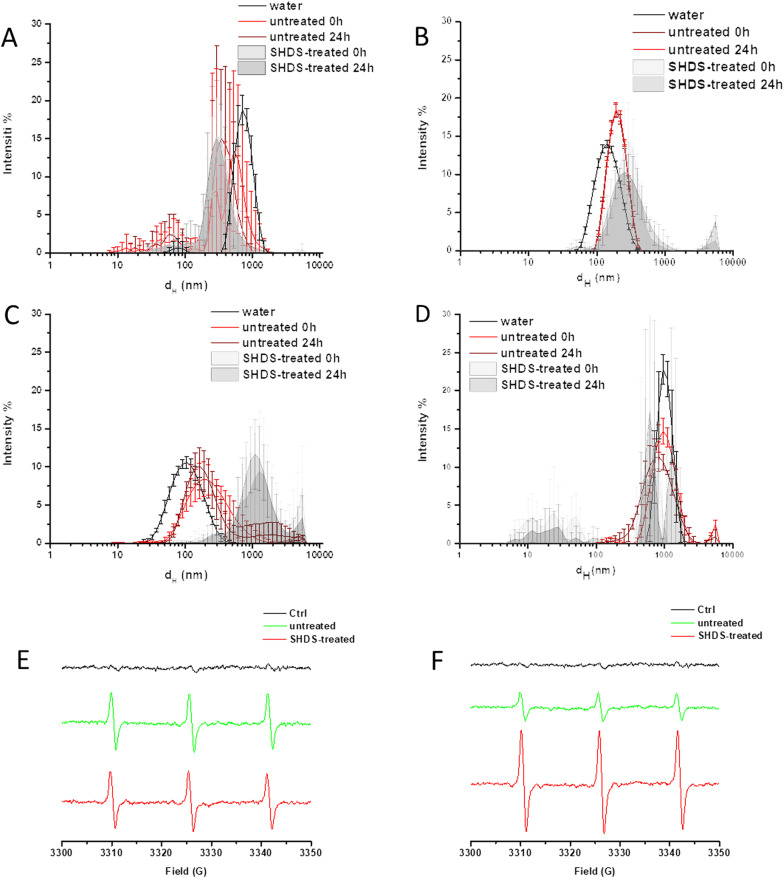
Identity of NBMs in cell medium. Upper panels: Comparison of the size distribution (DLS) of untreated and SHDS-treated NBMs in DMEM 10% FBS. **A** LSNPs; **B** CNPs; **C** FNPs; **D** HNPs. Lower panels: representative EPR spectra recorded in a suspension of **E** CNPs; **F** FNPs pre-incubated in DMEM 10% FBS in the presence of the spin-probe TEMPONE-H

In vitro cellular tests require the use of cell medium, which contains several components, including proteins. During the NBM incubation in this medium, their biological identity may be further modified due to the particles interaction with the medium components. Therefore, we firstly investigated any changes in the material size distribution following exposure to protein rich fluid. Fig  6 shows a comparison of treated and untreated NBMs diluted in cell medium (Dulbecco's Modified Eagle Medium (DMEM) with 10% Foetal Bovine Serum (FBS)). The mean d_H_ values and PDI are reported in Additional file 1: Table S1.

The NBMs exposure to the SHDS resulted in a dramatic change of the size distribution for all NBMs.

Treated LSNPs exposed to cell medium were more stable over time than the untreated ones (Fig. 6A), but they displayed a wider range of size, with a population characterised by a mean diameter smaller than the LSNPs in water, likely as a consequence of a partial degradation. Both treated and untreated CNPs were stable in cell medium up to 24 h (Fig. 6B). A moderate shift of the sizes towards values higher than the particles in water was however observed, more evident for the treated CNPs. A visible time-dependent instability in cell medium was observed for both treated and untreated FNPs (Fig. 6C) and HNPs (Fig. 6D). Treated FNPs were largely aggregated in cell medium, while a moderate shift of the curve towards higher d_H_ was observed for the untreated FNPs in comparison to water. Untreated HNPs appeared to form slightly more stable colloids in cell medium than in water; while after SHDS several populations with a wide range of size appeared. The formation of aggregates of size above the detection limit of DLS was clearly visible for all NBMs in cell medium (Additional file 1: Fig S5).

The surface reactivity of CNPs and FNPs in the cell medium was also monitored by EPR spectroscopy (Fig. 6E and F). Both treated and untreated CNPs had similar surface reactivity in cell medium, suggesting that the surface of the particles is still exposed to the solvent. In the case of FNPs, the SHDS-treated sample exhibited an unexpectedly high surface reactivity, likely caused by the presence in the bio-molecular corona of some redox-active components.

**Fig. 7:**
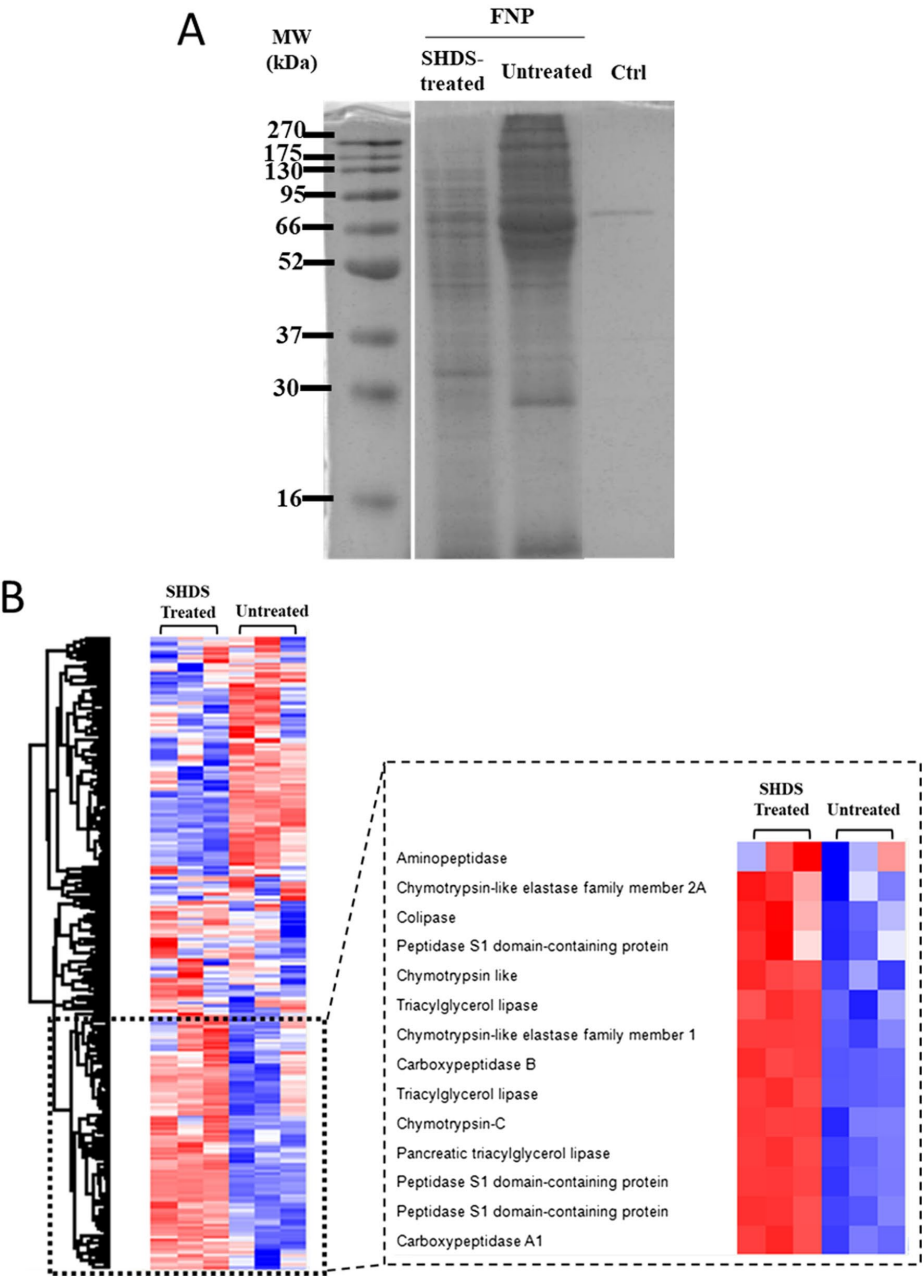
Effect of the SHDS on the protein corona composition of FNPs incubated in DMEM 10% FBS for 24 h. **A** SDS-PAGE analysis (Ctrl = SHDS + DMEM 10% FBS without FNPs). **B** Heat map showing the relative abundance for each protein normalizing the mean value for the same protein. Proteins derived by SHDS are highlighted in the square

The hard protein corona composition was also analysed for treated and untreated NBMs after incubation in cell medium. For the isolation of the corona-NBM complex, three steps of centrifugation were necessary to remove the soft corona [63]. However, a protein background was detected for the control sample, only composed by SHDS and DMEM 10% FBS and no NBMs (Additional file 1: Fig S6E), suggesting that the digestion process and the long incubation time led to protein aggregation. Unfortunately, it was not possible to discriminate the protein corona from the background proteins that co-precipitated during the NBMs-corona isolation protocol. Thus, we studied the effect of the treatment with SHDS only for FNPs. In fact, for these NBMs a magnetic separation was used as an alternative method to remove the soft corona. In Fig. 7A the SDS-PAGE analysis of the hard corona is shown. Clearly, the treatment with SHDS affected the corona composition since treated and untreated samples had different protein patterns.

Proteomic analysis by mass spectrometry for these samples identified more than 200 proteins for the corona of both SHDS-treated and untreated FNPs (Fig. 7B).

The top 20 most abundant proteins are listed in Table 2. Label free quantification (LFQ) calculated with Perseus was used to compare the protein abundance between the two samples.

**Table 2:** Top 20 most abundant proteins for SHDS-treated and untreated FNPs

	FNP SHDS + DMEM 10% FBS				FNP DMEM 10% FBS			
	LFQ ± SEM (x1E08)	Protein Name	Protein ID	MW (kDa)	LFQ ± SEM (x1E08)	Protein Name	Protein ID	MW (kDa)
1	12.4 ± 3.4	Bovine Alpha-2-HS-glycoprotein*	P12763	38.4	8.11 ± 1.6	Bovine Haemoglobin foetal subunit beta*	P02081	15.9
2	12.3 ± 1.8	Bovine Alpha-1-antiproteinase*	P34955	46.1	4.81 ± 0.19	Bovine Alpha-1-antiproteinase*	P34955	46.1
3	10.0 ± 0.90	Pig Peptidase S1 domain-containing protein**	I3LHI7	27.7	3.99 ± 0.050	Bovine Alpha-2-HS-glycoprotein*	P12763	38.4
4	7.96 ± 0.32	Bovine Albumin*	P02769	69.3	3.27 ± 0.34	Bovine Albumin*	P02769	69.3
5	6.84 ± 1.1	Bovine Haemoglobin foetal subunit beta*	P02081	15.9	3.22 ± 0.70	Bovine Haemoglobin subunit alpha*	P01966	15.2
6	4.03 ± 0.60	Bovine Apolipoprotein A-I*	P15497	30.3	2.67 ± 0.35	Bovine Apolipoprotein A-I*	P15497	30.3
7	3.42 ± 0.76	Bovine Histone H2A type 2-C	A1A4R1	14	2.45 ± 0.11	Bovine Inter-alpha-trypsin inhibitor heavy chain H2*	A0A3Q1LK49	96.8
8	2.45 ± 0.24	Bovine Haemoglobin subunit alpha*	P01966	15.2	2.35 ± 0.35	Bovine Angiotensinogen*	P01017	51.4
9	2.43 ± 0.35	Pig Triacylglycerol lipase**	F1S4T9	51.6	2.04 ± 0.18	Bovine Alpha-fetoprotein*	Q3SZ57	68.6
10	2.42 ± 0.35	Pig HATPase_c domain-containing protein**	A0A287A9T4	83	1.90 ± 0.16	Bovine Apolipoprotein A-II*	P81644	11.2
11	2.32 ± 0.57	Bovine Serpin family G member 1	E1BMJ0	51.8	1.88 ± 0.14	Bovine Inter-alpha-trypsin inhibitor heavy chain H4	F1MMD7	101.5
12	2.26 ± 0.42	Pig Carboxypeptidase A1**	P09954	47.2	1.87 ± 0.10	Bovine Alpha-1-microglobulin	F1MMK9	53
13	2.02 ± 0.80	Bovine Angiotensinogen*	P01017	51.4	1.80 ± 0.17	Bovine Beta-2-glycoprotein 1*	P17690	38.3
14	2.00 ± 0.13	Bovine Alpha-fetoprotein*	Q3SZ57	68.6	1.54 ± 0.090	Bovine Alpha-2-macroglobulin	Q7SIH1	167.6
15	1.61 ± 0.85	Bovine Histone H2A	F2Z4G5	14.1	1.52 ± 0.15	Bovine Fetuin-B	Q58D62	42.7
16	1.58 ± 0.10	Bovine Beta-2-glycoprotein 1*	P17690	38.3	1.46 ± 0.24	Bovine Vitronectin*	Q3ZBS7	53.6
17	1.58 ± 0.10	Bovine Inter-alpha-trypsin inhibitor heavy chain H2*	A0A3Q1LK49	96.8	1.46 ± 0.037	Bovine Complement C3	Q2UVX4	187.3
18	1.39 ± 0.16	Bovine Apolipoprotein A-II*	P81644	11.2	1.39 ± 0.12	Bovine Alpha-1B-glycoprotein	Q2KJF1	53.6
19	1.30 ± 0.19	Pig Peptidase S1 domain-containing protein**	I3LJ52	26.9	1.28 ± 0.11	Bovine Inter-alpha-trypsin inhibitor heavy chain H3	P56652	99.6
20	1.14 ± 1.7	Bovine Vitronectin*	Q3ZBS7	53.6	1.28 ± 0.11	Bovine Complement factor B	P81187	85.4

^*^Common proteins; **proteins from SHDS. LFQ was calculated with Perseus (*n* = 3). SEM refer to the standard error of the mean for *n* = 3

Within the top 20 most abundant proteins, some serine proteases derived from SHDS were found in the SHDS treated sample. Different chymotrypsins derived from pancreatin were also detected among the less abundant proteins (data not shown). On the other hand, proteins highly abundant in FBS [64], were found in the corona for both samples, in particular alpha-2-HS-glycoprotein, apolipoprotein AI and AII, bovine haemoglobin alpha and beta chain, and alpha-1-antiproteinase. This latter, alpha-1-antiproteinase, also known as alpha-1-antitrypsin, alpha-1-proteinase inhibitor or serpin A1, is an inhibitor of serine proteases. A comparison of the abundance of proteases in treated and untreated samples is shown in Additional file 1: Fig S7.

#### Effects of SHDS on the cytotoxicity toward epithelial intestinal Caco-2 cells, HCT116 cells and primary human colonic epithelial cells

To investigate the effect of SHDS treatment on the NBMs cytotoxicity, a dose-dependent viability assay in Caco-2 and HCT116 cells, using a dose range from 0 to 150 µg/ml was performed.

This range was chosen based on the toxicity given by the OGI fluids alone tested at the same dilutions used for NBMs. Indeed, preliminary experiments indicated that OGI fluids were not significantly toxic up to dilution corresponding to 150 µg/ml NBMs (Additional file 1: Fig S8A).

**Fig. 8:**
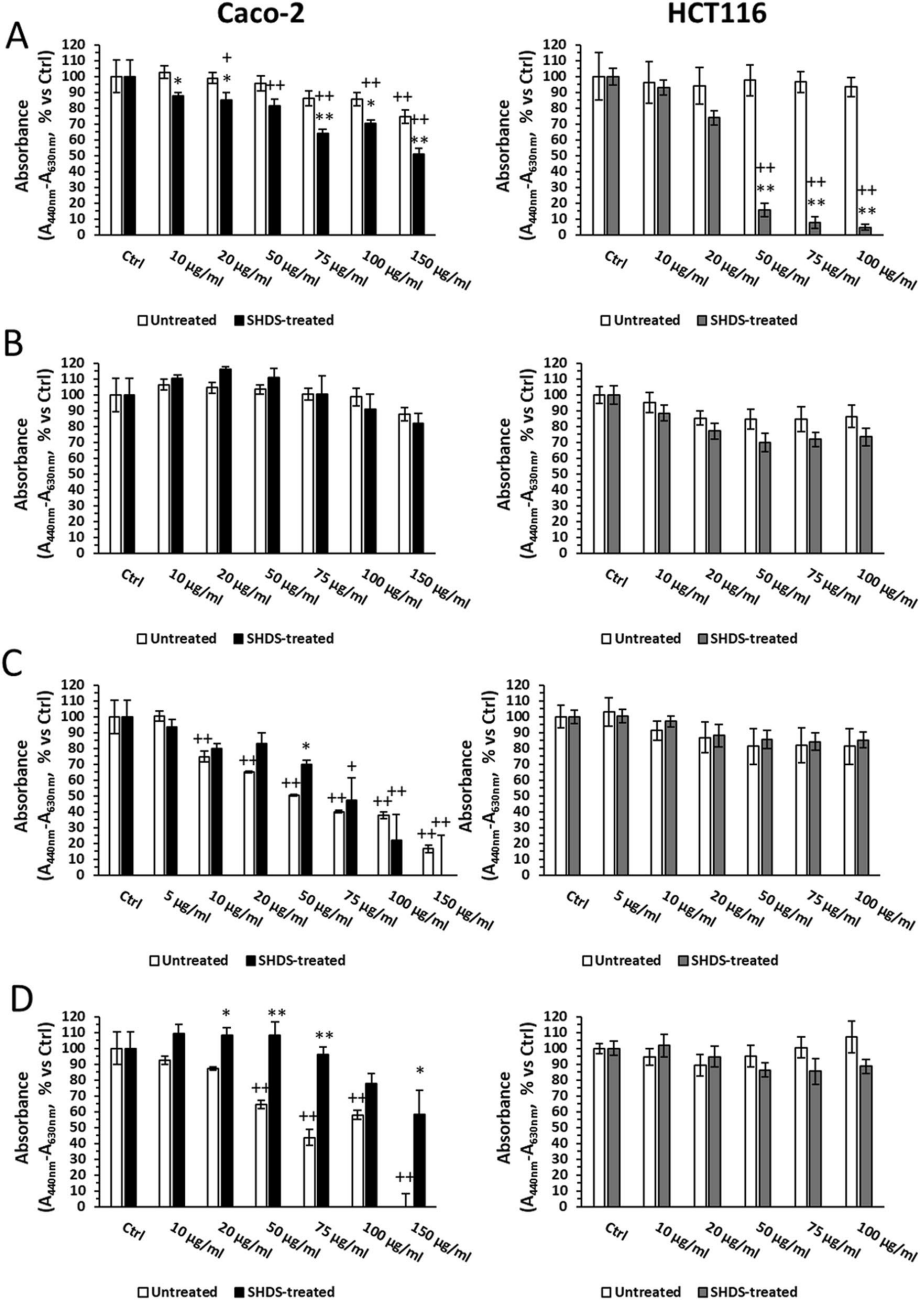
Cell viability of Caco-2 (left) and HCT116 (right) cells after incubation with SHDS treated and untreated **A** LSNPs, **B** CNPs, **C** FNPs and **D** HNPs. *n* = 3; mean ± standard error (SEM); ^+^*p* < 0.05 versus Ctrl; ^++^*p* < 0.01 versus Ctrl; *p < 0.05 versus untreated; **p < 0.01 versus untreated

In both Caco-2 and HCT116 cells, a very low toxicity was observed for untreated LSNPs up to 100 µg/ml. However, after the treatment with SHDS a significant cytotoxicity was observed for concentrations higher than 75 µg/ml and 20 µg/ml in Caco-2 and HCT116 cells, respectively (Fig. 8A). On the contrary, neither SHDS-treated nor untreated CNPs were toxic and SHDS did not alter the profile of toxicity of this NBM in both cell lines (Fig. 8B). On Caco-2 cells, untreated FNPs displayed the highest cytotoxicity, starting at 10 µg/ml, but in contrast with the other NBMs, SHDS has a cytoprotective effect: indeed, SHDS-treated FNPs became toxic at 50 µg/ml and were significantly less toxic than untreated ones in the range 50–75 µg/ml (Fig. 8C). A similar effect of toxicity masking was observed for HNPs: indeed, untreated HNPs were toxic from 50 µg/ml, but after SHDS no toxicity was detected at all the concentrations tested. As further confirmation, SHDS-treated HNPs were significantly less cytotoxic than untreated ones (Fig. 8D). On HCT116 cells, neither untreated nor SHDS-treated FNPs and HNPs induced any significant cytotoxicity (Fig. 8C and D).

**Fig. 9:**
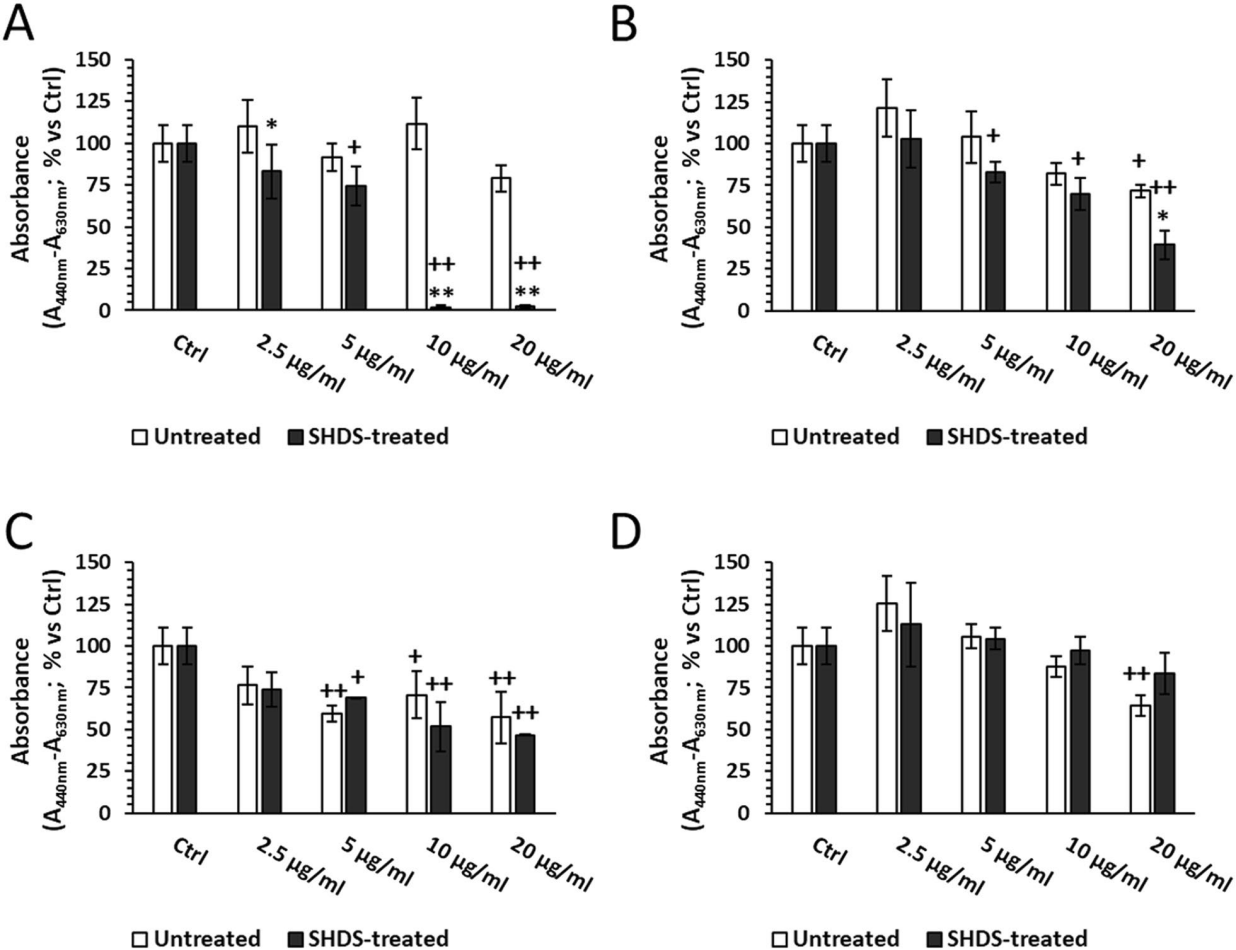
HCoEpiC viability after incubation with SHDS-treated and untreated **A** LSNPs, **B** CNPs, **C** FNPs and **D** HNPs. *n* = 3; mean ± SEM; ^+^*p* < 0.05 versus Ctrl; ^++^*p* < 0.01 versus Ctrl; *p < 0.05 versus untreated; **p < 0.01 versus untreated

Caco-2 and HCT116 cells are widely used as models of gastrointestinal cells [65]. However, being immortalized, they are expected to be more resistant to external stimuli. Therefore, we measured the effect of NBMs also in primary non-transformed intestinal epithelial cells (HCoEpiC). In general, all the NBMs showed toxicity at lower concentrations than on Caco-2 and HCT116 cells (Fig. 9). This finding can be partially explained by the higher sensitivity of HCoEpiC to SHDS fluids, which are toxic at a lower concentration (50 µg/ml) (Additional file 1: Fig S8B) than in immortalized cells (Additional file 1: Fig S8A). On this basis, we decided to evaluate the NBMs at a concentration range (2.5–20 µg/ml) immediately below the first toxic concentration for SHDS fluids.

The pre-incubation with SHDS increased the cytotoxicity of LSNPs and CNPs (Fig. 9A and B). We did not detect any significant cytotoxicity changes between SHDS-treated and untreated FNPs (Fig. 9C), while HNPs were the only NBM showing lower cytotoxicity after SHDS towards both Caco-2 (Fig. 8D) and HCoEpiC cells (Fig. 9D).

These data show a different behaviour on epithelial intestinal cells in relation to the nature of NBMs that can undergo different modifications during SHDS. Moreover, the choice of in vitro model to evaluate cytotoxicity is also of paramount importance, as demonstrated by the different sensitivity between Caco-2 cells, HCT116 cells and primary non-transformed cells.

The genotoxicity of NBMs was then evaluated on HCT116 cells because, although it is a cancer-derived cell line, it bears wild-type p53 contrarily to Caco-2 cells. None of the tested NBM induced any significant increase of DNA strand breaks as assessed by counting 53BP1 DNA repair foci, while the positive control, i.e., cells exposed for 24 h to 50 µM etoposide, led to a statistically significant increase of 53BP1 foci count (Additional file 1: Fig S9).

#### Effects of SHDS on viability and permeability of Caco-2 intestinal barrier model

Finally, we investigated the effects of NBMs on viability, permeability, and inflammation parameters in a competent model of GI barrier, i.e. the 21-day differentiated Caco-2 model.

When Caco-2 cells grow on specific inserts and reach the complete confluence, they begin to differentiate, completing the process after 21 days [66]. This model is recognized as a valid GI barrier model, widely used in permeability assessment tests because it mimics intestinal physiology [67, 68]. Thus, we used this model to investigate the effects on cell viability and barrier permeability for all SHDS-treated and untreated NBMs. Since there are no literature data about the physiological doses in patients exposed to these NBMs but at the same time there are also no reports of severe acute toxicity, we decided to study the highest non-toxic dose of NBMs for Caco-2 undifferentiated cells, to highlight the differences in terms of toxicological properties of NBMs, before and after the simulated digestive process. Barrier-forming cells were thus incubated for 24 h at the highest non-toxic concentrations of SHDS-treated NBMs (150 µg/ml for CNPs and HNPs and 50 µg/ml for LSNPs and FNPs) (Fig. 8, left panel).

**Fig. 10:**
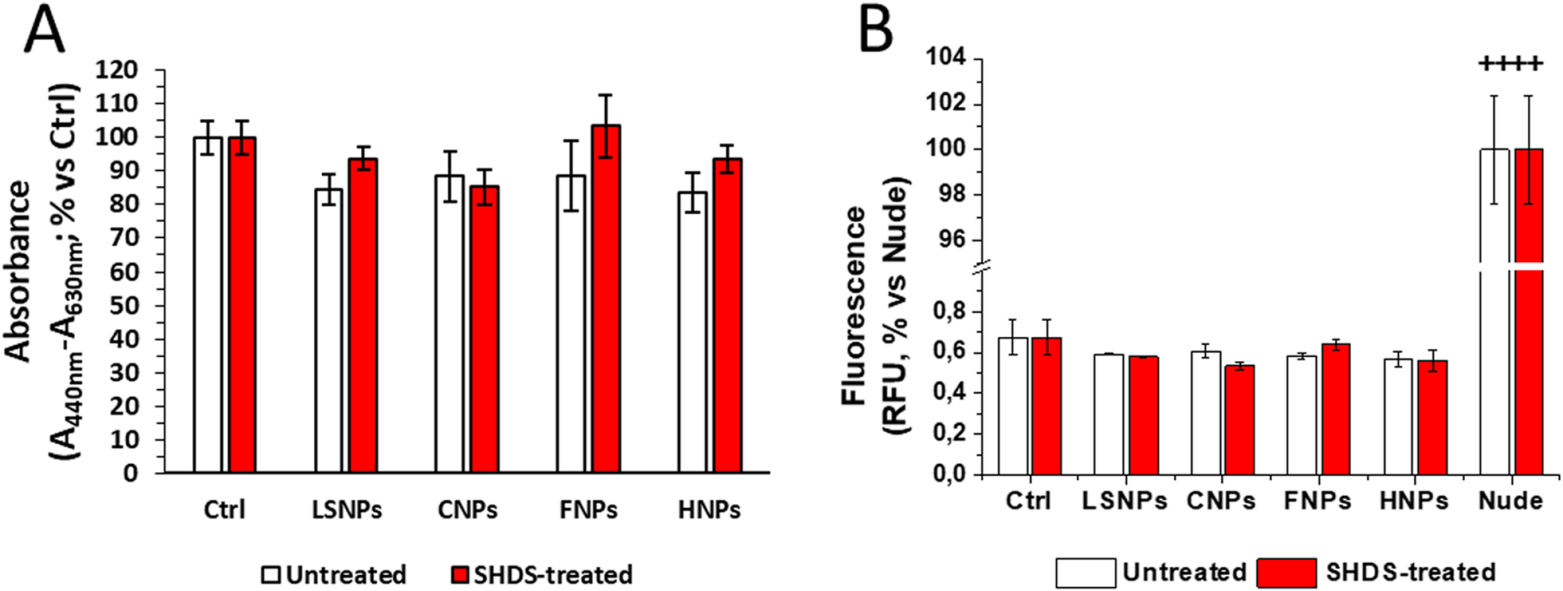
Caco-2 barrier viability (**A**) and permeability (**B**). 24 h of incubation with 50 µg/ml of LSNPs and FNPs, and 150 µg/ml of CNPs and HNPs. *n* = 3. Nude: permeability of Lucifer Yellow across Millicell® inserts without cells. *n* = 3; mean ± SEM; ^++^*p* < 0.01 versus Ctrl

In the viability assays, neither untreated nor SHDS-treated NBMs showed toxicity (Fig. 10A). Since toxic effects of NBMs may alter barrier integrity and produce inflammation [30], we next evaluated integrity parameters, in terms of functional assays, TEER and TJ levels, and pro/anti-inflammatory cytokines production.

In our model, the absence of toxicity was paralleled by the absence in permeability variation, measured by the Lucifer Yellow permeability assay (Fig. 10B; Table 3). Moreover, the TEER values were always > 600 Ω*cm^2^ in both untreated and NBM-treated barriers (Additional file 1: Table S2).

**Table 3:** Apparent permeability (Papp) values of Caco-2 barrier model after 24 h of incubation

	Untreated Papp (cm/s)	SHDS-treated Papp (cm/s)
Ctrl	2.61 × 10^–7^ ± 2.71 × 10^–8^	2.67 × 10^–7^ ± 1.40 × 10^–8^
LSNPs	2.32 × 10^–7^ ± 5.60 × 10^–9^	2.26 × 10^–7^ ± 3.20 × 10^–9^
CNPs	2.38 × 10^–7^ ± 1.83 × 10^–8^	2.09 × 10^–7^ ± 3.37 × 10^–9^
FNPs	2.29 × 10^–7^ ± 1.08 × 10^–8^	2.50 × 10^–7^ ± 1.35 × 10^–8^
HNPs	2.21 × 10^–7^ ± 9.92 × 10^–9^	2.18 × 10^–7^ ± 1.45 × 10^–8^

24 h of incubation with 50 µg/ml of LSNPs and FNPs, and 150 µg/ml of CNPs and HNPs. *n* = 3; mean ± SEM

**Fig. 11:**
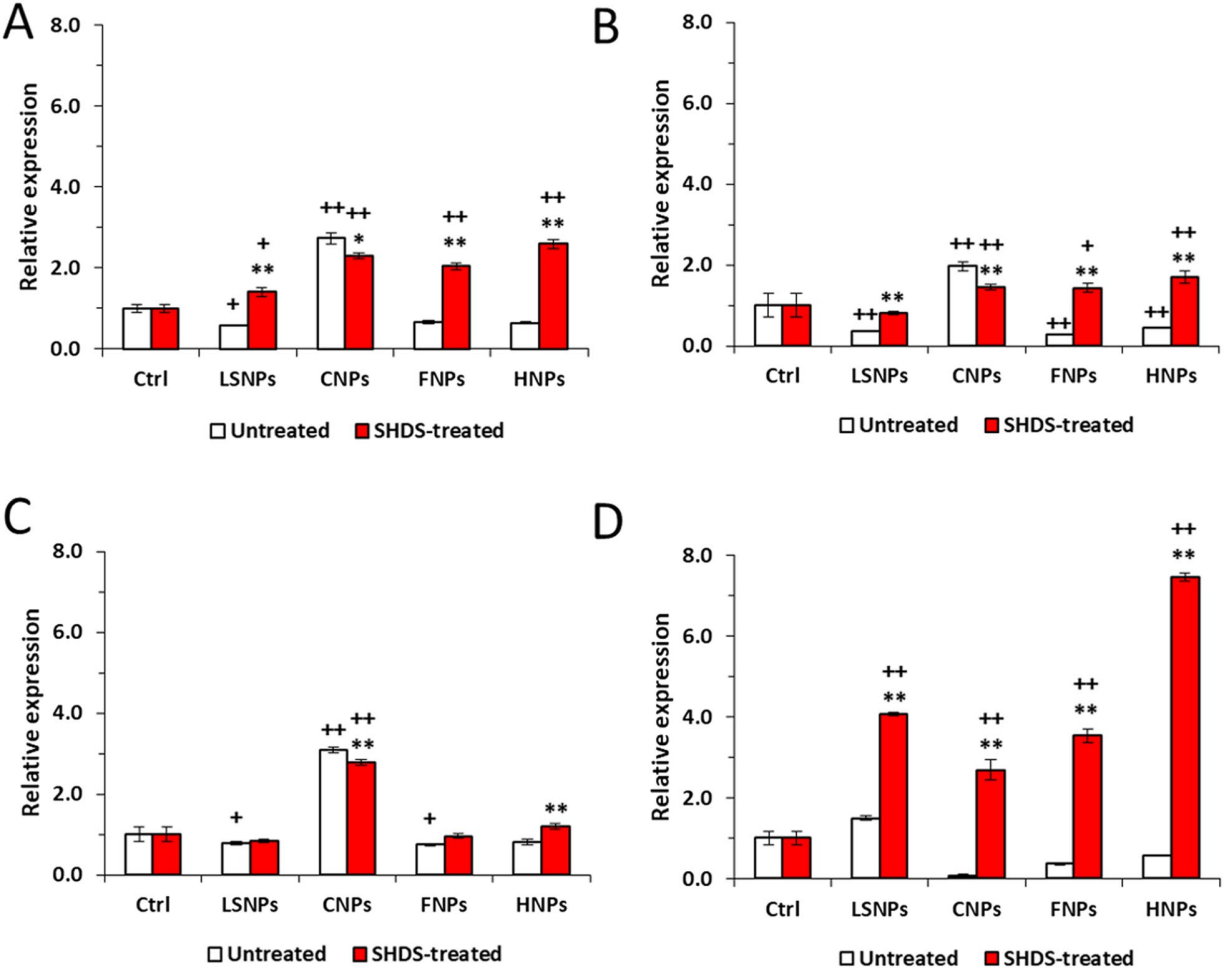
Tight junction proteins gene expression in Caco-2 barrier model after 24 h of incubation with 50 µg/ml of LSNPs and FNPs, and 150 µg/ml of CNPs and HNPs. **A** TJP1, **B** OCLN, **C** CLDN3 and **D** CLDN5. *n* = 3; mean ± SEM; ^+^*p* < 0.05 versus Ctrl; ^++^*p* < 0.01 versus Ctrl; *p < 0.05 versus untreated; **p < 0.01 versus untreated

Notably, each NBM increased one or more genes involved in TJs with a specific pattern (Fig. 11). Untreated LSNPs, FNPs and HNPs down-regulate occludin (OCLN) (Fig. 11B) and to a lesser extent zonula occludens-1 (TJP1) (Fig. 11A), claudin 3 (CLDN3) (Fig. 11C) and claudin 5 (CLDN5) (Fig. 11D) genes. SHDS-treated LSNPs increased only TJP1 and CLDN5, SHDS-treated FNPs increased TJP1, OCLN and CLDN5, SHDS-treated HNPs increased the expression of all these genes, although to a different extent. Interestingly, CNPs were the only NBM that increased the expression of all TJs-encoding genes evaluated both in the untreated (except for CLDN5) and in the SHDS-treated form (Fig. 11).

This up-regulation of TJs genes may suggest a compensatory response mounted by GI cells in response to potentially cytotoxic NBMs. According to the functional results in terms of permeability (Fig. 10B), such response was successful in preventing the loss of barrier integrity.

Finally, we analysed the gene expression of TNF-α and IL-6, two pro-inflammatory cytokines involved in the pathogenesis of inflammatory bowel disease [69], opposed to IL-10, known for its immune-suppressive role in inflammatory bowel disease [70], and to IL-22, which triggers regeneration after intestinal injuries [71] and preserves the intestinal epithelial integrity [72].

**Fig. 12:**
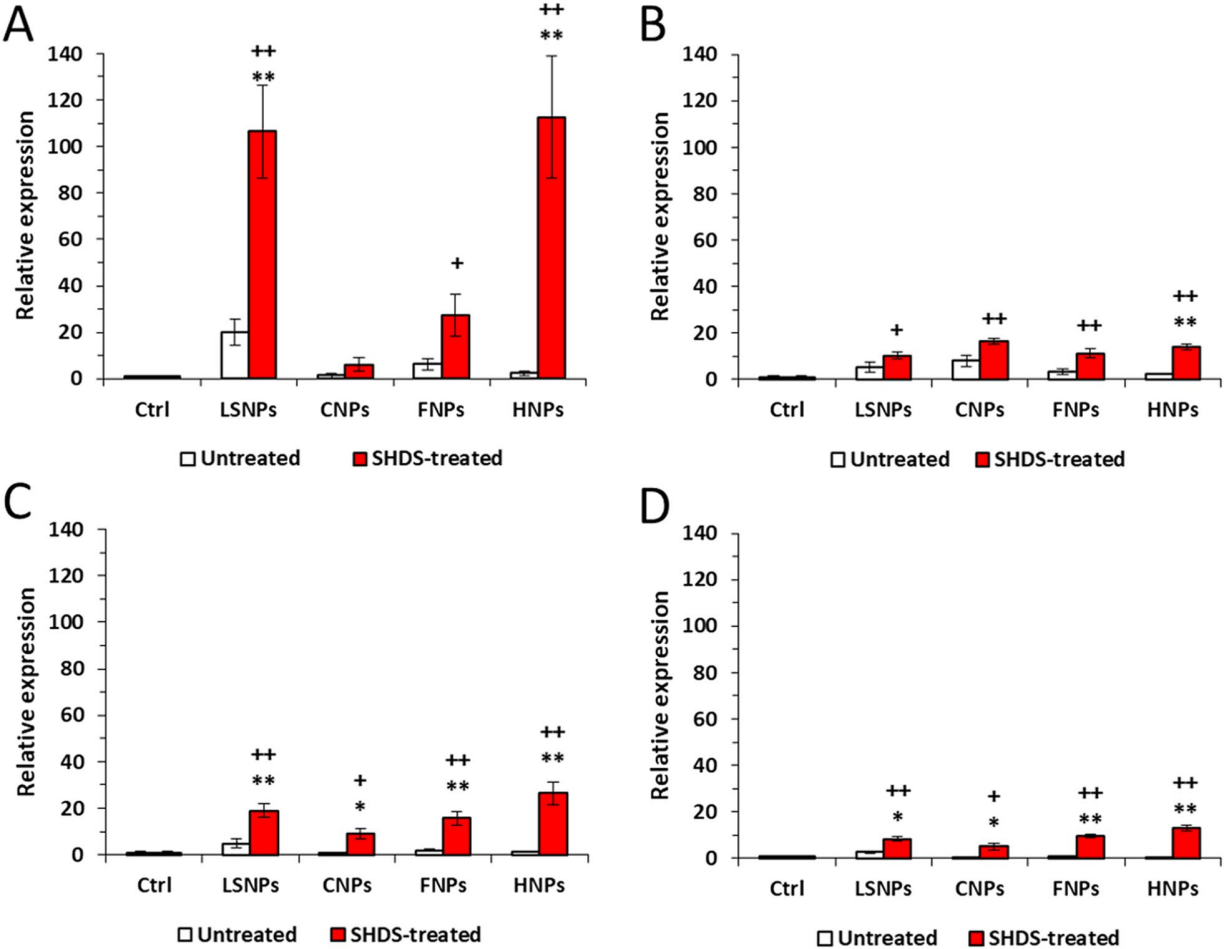
Inflammation gene expression in Caco-2 barrier model after 24 h of incubation with 50 µg/ml of LSNPs and FNPs, and 150 µg/ml of CNPs and HNPs. **A** TNF, **B** IL6, **C** IL10 and **D** IL22. *n* = 3; mean ± SEM; ^+^*p* < 0.05 versus Ctrl; ^++^*p* < 0.01 versus Ctrl; *p < 0.05 versus untreated; **p < 0.01 versus untreated

None of the untreated NBMs significantly increased the cytokines gene expression (Fig. 12), in line with the low modulation of TJ-genes (Fig. 11). SHDS-treated CNPs did not increase the expression of pro-inflammatory TNF, which was instead increased by SHDS-treated LSNPs, FNPs and HNPs (Fig. 12A). All the SHDS-treated NBMs increased IL6 (Fig. 12B), but they also up-regulated the anti-inflammatory/immune-suppressive cytokines IL10 (Fig. 12C) and IL22 (Fig. 12D), suggesting a balance between pro-inflammatory and anti-inflammatory processes that could contribute to preserve the GI barrier integrity.

#### NBM intestinal barrier crossing

The results obtained with the Lucifer Yellow test suggested that the tested NBMs are unable to cross the barrier, even after SHDS-treatment. To monitor the NBM possible transcellular translocation, the barrier model was exposed to CNPs and FNPs and the particle concentration in the basolateral compartment of Caco-2 GI barrier was monitored by Nanoparticle Track Analysis (NTA). These samples were selected because the technique is more sensible on samples with a highly refractive index. No particles were found in the basolateral compartment after treatment with both SHDS-treated and untreated CNPs and FNPs (data not shown). These data were confirmed for FNPs using the FerroZine™ assay which showed no significant presence of iron (data not shown).

## Discussion

The exposure to NBMs is becoming more and more common due to their widespread use in various industrial sectors including food and medicine. Consequently, the number of studies focusing on NBMs hazard are increasing exponentially [73–75]. Ingestion has been recognized as an important route of exposure to both nanomaterials and NBMs only recently. For this reason it has been little investigated so far. Moreover, because of the complexity of the OGI tract physiology, a consensus on the most suitable models and markers for the assessment of NBM toxicity has not been reached yet.

Recently, several cellular models have been proposed to mimic the manifold gut anatomy and physiology [32, 33, 76–79]. However, in most of the existing studies cells are exposed to untreated NBMs, neglecting the transformations that occur to NBMs during the transit in the OGI tract [13]. Recently, different in vitro systems simulating digestion have been proposed to monitor such biotransformation [50, 80, 81]. Nevertheless, few studies have been published on the impact that the NBM biotransformation along the OGI tract may have on their toxicity toward intestinal cells [39, 52–54, 82].

In the present study we found that the application of an in vitro Simulated Human Digestion System (SHDS) induces a significant modification of the bioidentity of four NBMs, which in turn modulates their bioactivity towards intestinal epithelial cells. Samples representative of NBMs with potential applications in oral drug delivery (FNPs, CNPs, HNPs, LSNPs) [55–58] or as ingredients of nutraceutical formulations have been selected (LSNPs) [83].

### NBMs acquire a new identity in the OGI tract

The concept that the NBM bioactivity strongly depends upon their physical and chemical properties is currently well consolidated. Size and surface properties are the parameters which have been recognized to modulate the NBM toxicity [84–86].

Size primarily affects dosimetry and cellular uptake. Smaller particles can penetrate cells more easily by active processes such as caveolae- and clathrin-mediated endocytosis, or by passive diffusion across the cell membrane [87]. On the other hand, size affects particle sedimentation and diffusion, thus modifying the kinetics of contact with cells and the effective dose [88, 89].

An increase of the particles size consequent to aggregation or agglomeration following contact with simulated gastric or intestinal fluids has been previously reported for several types of NBMs, as lipid nanoparticles [90], Ag nanoparticles [52, 91, 92], TiO_2_ nanoparticles [39, 93], silica nanoparticles [52, 94], amorphous Mg-Ca phosphate nanoparticles [95], Au nanoparticles [96], and others [97, 98]. Our study confirms previous reports, since a significant increase of the size has been observed for all the NBMs investigated (Fig. 2). More importantly, we demonstrate that this process, occurring mainly in the gastric compartment, is irreversible regardless of the chemical composition of the NBMs. In fact, all NBMs appear aggregated in the cell media, and in these forms have a higher probability to interact with the intestinal cells in vivo. The low pH and the high ionic strength of the gastric fluid are the main driving force of aggregation, as demonstrated for stabilized zero-valent iron nanoparticles [99] and Au nanoparticles [100], while proteins in the medium appear to partially inhibit the process.

However, aggregation is not the only transformation process that NBMs undergo. In fact, in the case of HNPs, LSNPs and FNPs the aggregates appear composed of particles smaller than the untreated ones. Dissolution, enzymatic degradation or coating degradation were observed, in line with other studies reporting that extreme pH can dissolve pH-sensitive NBMs [101], and that enzymes and proteins can contribute to NBMs dissolution by digesting NBMs components [102].

Another important aspect is the modification of the NBMs surface chemistry. Surface charge both affects colloidal stability and nanoparticles-membrane interaction [103–105]. On the other hand, the NBM surface acts as a scaffold that binds proteins and biomolecules, leading to the acquisition of a new biological identity [106–110] that influences the NBMs affinity for different cell types and specific receptors [51, 87]. The composition of bio-molecular corona and subsequent NBMs activity strongly depend on the specific bio-fluid in which they are dispersed [111–114].

Our study clearly shows that proteins and other components (e.g., bile salts) can irreversibly bind to the surface by forming a hard corona. Clear-cut differences in the bio-molecular corona were found between untreated FNPs and SHDS-treated FNPs. Indeed, SDS-PAGE and mass spectrometry identified different proteases derived from SHDS. This corona also partially protects HNPs and LSNPs from degradation. The protective effect of proteins has been previously observed for hydroxyapatite nanoparticles after the addition of milk to the in vitro digestive process, resulting in a delayed dissolution due to the proteins coating [115]. Moreover, Levak et al., and Martin et al. demonstrate a minor release of Ag^+^ ions by silver nanoparticles when coated with proteins [116, 117]. On the other hand, the bio-molecular corona modifies the surface charge, but does not completely inhibit the surface reactivity of CNPs and FNPs, suggesting that the surface of the particles is still partially exposed to the solvent, and can interact directly with the cells. This effect is clearly dependent on the nature of the materials. Indeed, we have previously reported that in the case of TiO_2_ the treatment with the SHDS completely inhibited the surface reactivity [39]. These results underline the importance of the presence of active components in SHDS to accurately describe the NBMs transformation.

### Effect of the biotransformation on the toxicity of NBMs toward intestinal cells

The pre-treatment with the SHDS largely affected the behaviour of NBMs toward Caco-2 cells. However, the effects were different, depending on the type of NBMs and on the model/endpoint. The treatment increased the toxicity of LSNPs toward undifferentiated Caco-2 cells, likely because of the degradation of the outermost layers’ and the release of the surfactants, induced by the SHDS. On the contrary, the SHDS did not change the cytotoxicity of CNPs, and reduced the cytotoxicity of FNPs and HNPs (Fig. 8). The trend was different in primary epithelial intestinal cells. In this case, SHDS increased the toxicity of CNPs and FNPs, while LSNPs and HNPs maintained a toxicity trend like those observed in Caco-2 cells (Fig. 9). Overall, the primary cells appeared to be more sensitive to homeostatic perturbations. This finding is in line with other non-transformed cell lines as CCD-841, which showed a stronger decrease in viability after treatment with isothiocyanate-capped silicon nanoparticles in comparison with Caco-2 cells [118]. Our results suggest that the use of primary epithelial cells helps in obtaining a more complete picture of the effects of NBMs in humans, integrating the results obtained on immortalized cells, useful for preliminary and large-scale screenings, with the results obtained in a model closer to the cells of the human GI tract. However, we recommend to use both these models in parallel, in order to obtain multiple information at the same time: if on the one hand, primary cells gives more reliable information on the toxicity outcome in non-transformed gastro-intestinal tissue, on the other hand the use of immortalised cells that can form a competent gastrointestinal barrier allows to obtain information about the impact of NBMs on the barrier integrity.

Interestingly, both treated and untreated NBMs were not cytotoxic in the differentiated Caco-2 cells and did not alter the permeability of the intestinal barrier, while a clear perturbation of the TJs was observed for all NBMs. This may be interpreted as a compensatory mechanism: to limit the damage induced by NBMs, GI cells likely respond by increasing the expression of specific genes encoding for the main TJs proteins. By increasing the amount of TJs complexes, this response maintains the GI barrier intact, as it occurs as a compensatory mechanism in different diseases [119] or in response to IL-10, a cytokine increased by NBMs in our model and known to preserve the GI barrier integrity [29]. Indeed, while the pro-inflammatory cytokines TNF-α and IL-6, whose genes also are up-regulated by NBMs, are known to promote the disruption of the GI barrier [29], also IL-10 is concurrently over-expressed after the exposure to NMBs: this balance may promote the recovery of the barrier integrity after an initial inflammation-related damage. The induction of TJ protein genes was much more evident in SHDS-treated samples. This might be due to the presence of proteases derived from the SHDS in the hard corona, as demonstrated for FNPs. In fact, protease/antiprotease balance has been reported to be important in maintaining and regulating the intestinal permeability [120]. On the other hand, this effect could be compensated by the high presence of protease inhibitors derived from the cell media. This hypothesis should not necessarily apply to the other NBMs tested, since the protein corona composition is dependent on the chemical nature of the NBM. Conversely, it has been reported that TiO_2_ nanoparticles down-regulated TJs in vivo and ex vivo in mice, increasing paracellular permeability [121]. In some cases, it is the combination of NBMs as TiO_2_ or SiO_2_ with additives found in food [122] or with bacterial toxins as lipopolysaccharide [123] to reduce TJ, adherens junction and gap junction proteins [123]. Since no increase in the permeability of Lucifer Yellow was detected in our experimental conditions for all the NBMs tested, we concluded that the increase in TJs genes elicited by NBMs was sufficient to prevent any loss of GI barrier integrity. Alternatively, we cannot exclude that the integrity of the GI barrier that we measured was the result of a complete process of barrier reparation after an initial damage: indeed, it has been documented that Caco-2 cells exposed to silica nanoparticles undergo to an initial disruption of actin cytoskeleton and TJs architecture, followed by a recovery phase of actin remodelling and TJs reassembly [17]. We recognize that one limitation of our work is that we studied the acute effects only. Indeed, our main focus was the acute toxicity of NBMs, because the NBMs studied are used for medical purposes. Therefore, the exposure to the gastrointestinal cells is acute and not chronic. As follow up, we plan to monitor the TJ changes over time, in order to have a deeper insight into the time-dependent modulation of these parameters, focusing on NBMs whose exposure is chronic for environmental or occupational reasons.

As expected, both untreated and treated FNPs and CNPs did not cross the barrier by transcellular or paracellular routes.

Since NBMs are not-self components it has been widely reported that the exposure of GI barrier to NBMs induces local inflammation, supported by the presence of abundant lymphoid tissues associated with the intestinal mucosa [124] and/or by alterations in the gut microbiota [125]. Also, epithelial cells physiologically produce cytokines and chemokines that are critical in controlling the immune cells activation and the homeostasis of microbiota [126]. An altered production of cytokines from epithelial cells may result in dysbiosis, pathogenic infections or inflammatory bowel disease [126]. As final parameter of biocompatibility, we thus evaluated how the NBMs tested may alter the production of pro-inflammatory and anti-inflammatory cytokines by Caco-2 cells. The increase expression in typical pro-inflammatory cytokines as TNF and IL6, but also in anti-inflammatory cytokines as IL10 and in IL22, related to GI epithelial regeneration, elicited by NBMs may suggest the development of inflammatory events induced by NBMs exposure, paralleled by a compensatory secretion of anti-inflammatory and pro-regenerative cytokines. This balance, together with the over-expression of TJ genes, likely contributes to prevent barrier damage and integrity loss. Our data are in line with the work of Colombo and co-workers, reporting that commercial ZnO nanoparticles increase IL-6 and IL-8 production in Caco-2 barrier model, maintaining barrier integrity [127]. Similarly, polyvinyl chloride particles have been reported to induce IL-1β secretion without altering Caco-2/HT29-MTX/THP1 barrier integrity and viability [128]. All these works, however, do not consider the transformation that occurs during the NBM digestion. Indeed, in the case of SHDS-treated NBMs, we observed a significantly stronger increase of TJ and cytokine genes, likely because of the dramatic modifications experienced by the NBMs following the treatment with the SHDS. However, the lack of increased permeability in Caco-2 barrier exposed to SHDS-treated NBMs indicates that these modifications are coupled with preserved barrier integrity. Notably, CNPs had the lower effects on TJs and cytokines genes even after digestion, confirming themselves as the NBMs less modified during GI transit and more biocompatible after oral ingestion.

## Conclusions

In this work we developed a robust and consolidated pipeline that combines deep chemical-physical characterization techniques, microscopic analysis, simulated digestion and read-out of biological events, including biological assays on primary cells, to provide information on the toxicity in non-transformed gastro-intestinal tissue, and on barrier-forming immortalized cells, to obtain information about gastrointestinal barrier integrity and inflammatory events, after acute exposure to NBMs. Overall, the results add a piece of evidence on the importance of associating validated chemical and microscopic characterization, SHDS methods and in vitro models for the assessment of NBM intestinal acute toxicity and biocompatibility. Our pipeline is versatile, meaning that it can be applied to different NBMs that can be ingested accidentally, for environmental or occupational reasons. At the same time, it could provide a huge amount of information on NBMs transformation and acute effects on gastrointestinal tract cells. Further studies will be necessary to validate the reported results in vivo.

## Materials and methods

### Materials and reagents

Plasticware for cell cultures was from Falcon (Becton Dickinson, Franklin Lakes, NJ). FBS and culture medium were from Invitrogen Life Technologies (Carlsbad, CA). If not otherwise specified, reagents were purchased from Sigma-Merck.

### Nano-biomaterials (NBMs)

CNPs were synthesized by hydrothermal carbonization of glucose, following a protocol previously described [129]. CNPs are composed of elemental carbon, mainly amorphous, and are produced as colloidal suspension in water.

LSNPs, developed by Nanovector srl, Torino, Italy, are composed by water (Citrate/Phosphate buffer pH 5), glycerol, soy lecithin, glyceryl citrate/lactate/oleate/linoleate (E-472), glycerol monostearate (E-471), polysorbate 20, ascorbyl palmitate, sodium benzoate, α-tocopheryl acetate, strawberry flavour, sucralose and loaded with Melatonin (0.1% (w/w)). FNPs are a colloidal suspension in phosphate buffer (1 mM) of Fe_3_O_4_ nanoparticles embedded in a polymeric matrix (poly-lactic-co-glycolic acid/polyethylene glycol) developed by Colorobbia Consulting, Vinci, Italy.

HNPs have been purchased by Sigma Aldrich (Merck KGaA, Darmstadt, Germany) in the form of a powder made of pure hydroxyapatite with stoichiometric composition (Ca_5_(PO_4_)_3_OH).

### Dynamic light scattering (DLS)

Size distribution and polydispersity index (PDI) were measured on SHDS-treated and untreated NBMs diluted in ultrapure water (100 µg/ml) or in cell medium (DMEM supplemented with 10% FBS, 1% penicillin/streptomycin solution) (100 µg/ml). Measurements were performed by using the Zetasizer, Nano instrument (Malvern Instruments, Malvern, UK) with a 633 nm HeNe laser. Instrument settings were: replicate 3, equilibrium time 60 s, *T* = 25 °C, dispersant refractive index 1.330 (water) and 1.345 (cell medium), dispersant viscosity 0.8872 cP (water) and 0.8000 cP (cell medium), material refractive index 1.410 (LSNPs), 2.420 (CNPs), 2.420 (FNPs) and 1.650 (HNPs), material absorption 1.000.

Untreated CNPs, LSNPs and FNPs were diluted in ultrapure water before the analysis, while HNPs were suspended in ultrapure water and sonicated for 5 min with a probe sonicator (Sonoplus HD3100 Bandelin, Microtip MS73, diameter 3 mm, power 100 W, amplitude 30%). SHDS-treated NBMs were analysed in the fluids without dilution. In each experiment three subsequent measurements were performed on the same suspension. The data were expressed as the mean of three independent experiments, ± standard deviation. Each line represents the mean values of 15 measurements that were obtained in three independent experiments.

### Electrophoretic light scattering (ELS)

ζ-potential was measured using an electrophoretic light scattering analyzer (Zetasizer, Nano ZS Malvern Instruments, Malvern, UK). For ζ-potential curve versus pH, NBMs were diluted at 500 µg/ml in ultrapure water and pH was modified by adding NaOH 0.1 M or HCl 0.1 M. Instrument settings were: dispersant (water) dielectric constant: 78.5.

### Flow particle imaging analysis (FPIA)

FPIA was performed by using a Sysmex FPIA3000 analyser. High power field (2 × secondary lens) was applied, which allows measuring particles from 1 to 40 μm. The suspensions of nanoparticles in simulated digestive fluids were centrifuged at 8000 rpm for 10 min by Rotina 380 R (Hettich Zentrifuger). The suspensions were pelleted, the supernatant was removed, and the resulting pellets were resuspended in ultrapure water for 2 min in an ultrasonic bath. The washing process was repeated three times and 5 ml of the suspensions were analysed.

### Surface reactivity

The NBM surface reactivity was monitored by EPR analysis (Miniscope 100 EPR spectrometer, Magnettech, Berlin, Germany) using TEMPONE-H (1-hydroxy-2,2,6,6-tetramethyl-4-oxo-piperidine, Enzo Life Sciences, Inc.) as spin probe. Suspension of untreated or SHDS-treated NBMs in ultrapure water or cell medium (0.5 mg/ml) was diluted 1:1 in a 100 µM solution of Tempone-H and the suspension constantly stirred in a glass vial. The EPR spectra were recorded on a sample aliquot (50 µl). Instrument settings: microwave power 7 mW, modulation amplitude 1 G, scan time 80 s, two scans.

### Transmission electron microscope (TEM) measurements

TEM was accomplished utilizing a FEI CM20 microscope operating at 200 kV. TEM samples were prepared by placing one drop of a diluted sample on a carbon-coated Cu grid and allowing the solvent to evaporate.

### Protein corona analysis

NBMs were treated with SHDS following the protocol explained below. Treated and untreated NBMs were incubated for 24 h in DMEM 10% FBS, 1% penicillin/streptomycin at 37 °C under agitation (0.5 mg/ml). After the incubation, the NBM-corona complex was isolated through three centrifugation/dispersion cycles in PBS. For FNPs, ferromagnetic spheres were used to isolate the complex NBMs-corona (unpublished data). For SDS-PAGE, the pellets obtained after the washing were stripped using a loading buffer (Cell Signalling Technology) in 0.1 M dithiothreitol and heated at 100 °C. The obtained solutions were centrifuged before loading the samples in a 10% acrylamide gel. SDS-PAGE was carried out using the Mini-Protean (BioRad) system at 120 V until the dye front reached the end of the gel. The gels were stained using Coomassie (Thermo Scientific) and scanned with the Amersham Gel doc system.

For the mass spectrometry analysis, the samples in-gel were digested with trypsin and treated with different solutions to extract the peptides from the gel matrix. Raw mass spectrometry data were processed using the MaxQuant version 2.0.1.0. [130]. The identification of peptides and proteins was done using the UniProt database. Perseus software version 1.6.15.0 [131] allowed the analysis of the LFQ intensities obtained. Data were log transformed and missing values were replaced with values from a normal distribution.

### Cell cultures

Caco-2 epithelial colon cells were obtained from American Tissue Culture Collection (ATCC) and were grown in DMEM supplemented with 20% FBS, 1% penicillin/streptomycin. For the experiments, cells were used between passage 33 and 47, and incubated in DMEM supplemented with 10% FBS, 1% penicillin/streptomycin. To obtain Caco-2 monolayer forming a competent intestinal barrier model, cells were grown on Millicell®-96 cell culture inserts (Merck KGaA, Darmstadt, Germany) for 21 days [132].

HCT116 cells were obtained from the European Collection of Authenticated Cell Cultures (ECACC, catalogue No. #91,091,005) and were grown and exposed to NBMs in McCoy's 5a medium containing 2 mM glutamine, 10% FBS and 1% penicillin/streptomycin. Cells were used between passage 15 and 25.

Human colonic epithelial cells (HCoEpiC) were purchased from CliniSciences (CliniSciences, Guidonia Montecelio, Italy) and were cultured in Colonic Epithelial Cell Medium (HCoEpiCM) supplemented with 10% (v/v) Colonic Epithelial Cell Growth Supplement (HCoEpiCGS) and 1% penicillin/streptomycin. Experiments were performed between the passage 5 and 8.

### Preparation of simulated digestive fluids

Simulated digestive fluids were prepared following the protocol used by Sohal et al. [50]. The composition of each simulated digestive fluid is summarized in Table 4. For each fluid, the organic and inorganic parts were prepared separately by adding the components to ultrapure water and dissolving them under magnetic stirring. Then, the two solutions were mixed in a ratio of 1:1 (v/v) and stirred overnight.

**Table 4:** Composition of simulated digestive fluids for the SHDS model (amounts based on 100 ml of fluid)

Fluids	Saliva	Gastric juice	Duodenal fluid	Bile
pH	6.5 ± 0.1	1.4 ± 0.1	8.1 ± 0.1	8.0 ± 0.1
Inorganic fraction	89.6 mg KCl 20 mg KSCN 102.2 mg NaH_2_PO_4_xH_2_O 57 mg Na_2_SO4 29.8 mg NaCl Milli-Q water	30.6 mg NH_4_Cl 40 mg CaCl_2_ × 2H_2_O 82.4 mg KCl 275.2 mg NaCl 30.6 mg NaH_2_PO_4_xH_2_O Milli-Q water	5 mg MgCl_2_ × 6H_2_O 56.4 mg KCl 8 mg KH_2_PO4 338.8 mg NaHCO_3_ 701.2 mg NaCl Milli-Q water	37.6 mg KCl 578.5 mg NaHCO_3_ 525.9 mg NaCl Milli-Q water
Organic fraction	20 mg urea Milli-Q water	8.5 mg urea 65 mg D-glucose 2 mg glucuronic acid 33 mg D-glucosamine hydrochloride Milli-Q water	25 mg urea Milli-Q water	10 mg urea Milli-Q water
Active components	5 mg mucin (porcine stomach) 1.6 mg uric acid 14.5 mg α-amylase (Bacillus subtilis)	300 mg mucin (porcine stomach) 100 mg albumin (bovine serum) 100 mg pepsin (porcine gastric mucosa)	300 mg pancreatin (porcine pancreas) 50 mg lipase from (Candida rugosa) 100 mg albumin (bovine serum)	600 mg bile (bovine) 180 mg albumin (bovine serum)

The active components were added just before performing the experiment and the solution was vortexed to suspend them.

### Simulated human digestion system

The protocol used for the simulated human digestion system (SHDS) is that used by Sohal et al. [50].

A NBM suspension at the concentration of 1 mg/ml was SHDS-treated using an equal volume of simulated digestive fluids (Additional file 1: Fig S10). First, the SSF was added and the sample was incubated for 15 min at 37 °C under shaking. After this time, SGF was added and incubated for 4 h. Finally, SIF, composed of simulated duodenal fluid (SDF) and simulated bile fluid (SBF) in a ratio of 2:1 (v/v), was added and incubated for further 4 h. The ratio of simulated digestive fluids was 1:2:3 (Additional file 1: Fig S10). Table 4 summarises the simulated digestive fluids composition. At the end of the process, if necessary, pH was adjusted in the range of 6.5 and 7.5 using 1 M NaHCO_3_ and the suspension was sterilized 15 min under UV radiations.

For surface reactivity evaluation the suspension was centrifuged at 11,000 rpm (Rotina 380 R, Hettich Zentrifuger) and, after discarding the supernatant, it was resuspended in cell culture medium in a volume depending on the desired concentration.

For other tests the suspension was mixed with cell medium to obtain the final concentration. Incubation with cells or intestinal barrier model was performed for 24 h.

### Viability assay

WST-1 assay, based on the cleavage of the slightly red tetrazolium salt WST-1 (4-[3-(4-iodophenyl)-2-(4-nitrophenyl)-2H-5-tetrazolio]-1,3-benzene disulfonate) to form a dark red formazan dye by metabolically active cells, was used to evaluate the cell viability, as index of mitochondrial activity, after the treatment with NBMs. WST-1 was added at 10% (v/v) of cell medium and the absorbance was read at 440 nm after 2 h for Caco-2 cells, 1 h and 30 min for HCT116 cells, 4 h for HCoEpiC cells and 30 min for the intestinal barrier model using a Synergy HT Multi-Detection Microplate Reader (Bio-Tek Instruments, Winooski, VT) or a Spectramax ID3 plate reader (Molecular Devices, for HCT116 cells only). The absorbance value at the reference wavelength (630 nm) was subtracted.

### Genotoxicity assay

Genotoxicity was assessed by counting the DNA double strand break repair foci, after immunostaining of the 53BP1 DNA repair protein, as previously described [21, 133]. Briefly, after exposure to NBMs, cells were fixed with 4% paraformaldehyde, and permeabilized with 0.2% v/v Triton X-100 prepared in PBS containing 3% w/v bovine serum albumin (PBS-BSA). Non-specific sites were blocked with PBS-BSA, then incubated for 1 h with rabbit polyclonal anti-TP53BP1 antibody (Abnova, reference PAB12506) diluted in PBS-BSA, washed three times for 5 min with PBS-BSA and incubated for 1 h with an anti-rabbit IgG Atto 633 antibody (Sigma-Aldrich, 41,176) diluted in PBS-BSA. After three washing in PBS-BSA containing 0.2% Triton X-100, the nuclei were stained with 0.3 μg/ml Hoechst 33,342 (Sigma-Aldrich) for 20 min at room temperature. The number of cell nuclei and the average number of 53BP1 foci per cell nucleus were counted using a CellInsight CX5 High-Content Screening Platform (Thermo Fisher Scientific). This experiment was repeated three times independently, with *n* = 5 replicates in each independent experiment.

### Trans-Epithelial Electrical Resistance (TEER)

To evaluate the barrier formation and integrity of Caco-2 barrier model after the exposure to NBMs, TEER was measured using the Millicell® ERS-2 voltohmmeter (Merck KGaA, Darmstadt, Germany). The resistance was read in ohms and the resistivity was calculated by subtracting the cell-free inserts value from the cell-containing inserts, multiplying for the cells growth area. Only the monolayer with values > 250 Ω*cm^2^ were used for exposure to NBMs.

### Evaluation of barrier permeability

To evaluate the permeability of the intestinal barrier model the trans-epithelial passage of Lucifer Yellow fluorescent dye [134] was measured following the NANoREG SOP (Standard Operating Procedure for evaluation of NPs impact on Caco2 cell barrier model). After collection of the medium, the cells and the basolateral compartment of Millicell®-96 cell culture inserts were rinsed thrice with Hanks’ Balanced Salt Solution (HBSS). Then 50 µl/well of a 0.4 mg/ml Lucifer Yellow solution in HBSS were added in the apical compartment. After 2 h of incubation at 37 °C, the apparent permeability (Papp) and the percentage of fluorophore recovered from the lower chamber in cell-free inserts were calculated, reading the relative fluorescence units (RFUs) (λ excitation: 504 nm, λ emission: 529 nm), with a Synergy HT Multi-Detection Microplate Reader.

### NBMs absorption through the intestinal barrier by Nanoparticle Tracking Analysis (NTA)

The CNPs and FNPs passage through the Caco-2 intestinal barrier was evaluated by measuring the number of particles in the initial suspension and in the basolateral compartment of the Millicell®-96 cell culture inserts using Nanoparticle Tracking Analysis (NTA, ZetaView, Particle Metrix GmbH, Germany). The samples were diluted in ultrapure water before the analysis. Sensitivity was set at 60 and shutter value at 100. In this condition neither phenol red nor FBS interference was detected.

### FNPs absorption through the intestinal barrier by colorimetric assay

For the quantification of FNPs in the basolateral compartment the FerroZine™-based colorimetric assay was used based on the protocols reported by Balivada and co-workers [135] and by Jeinter [136]. First, to dissolve and reduce the iron contained in FNPs, 150 µl of the samples were incubated at 70 °C for 2 h with 150 µl of 1.2 M HCl and 60 µl of 1 M ascorbic acid. Then, 300 µl of the resulting solution were incubated with 200 µl of 1.5 M sodium acetate, 50 µl of 1 M ascorbic acid, 350 µl of ultrapure water, and 100 µl of 21 mM FerroZine™ (Thermo Fisher Scientific). After 20 min of incubation at room temperature, the absorbance at 562 nm was measured by an UV–Vis spectrophotometer (UVICON 930, Kontron Instruments, Basel, Switzerland) and the concentration was calculated using a calibration curve.

### Quantitative Real-Time PCR (qRT-PCR)

mRNA was extracted using the phenol/chloroform/ethanol method: cells were lysed in 0.5 ml RiboZol (VWR; Radnor, PA) and incubated for 10 min at room temperature, and then 0.2 ml chloroform was added. Samples were shaken for 15 s and incubated at room temperature for 3 min before being centrifuged for 15 min at 12,000*g* at 4 °C. Aqueous phase was transferred in a new tube and 0.2 ml isopropanol was added, the samples were incubated for 10 min at room temperature before centrifugation at 12,000*g* for 10 min at 4 °C. RNA pellets were washed twice with ethanol 70% v/v by centrifuging at 12,000*g* for 5 min at 4 °C, and were resuspended in RNAse-free water. The RNA quantification was performed using the Take3 plate (Synergy HT Multi-Detection Microplate Reader), reading the absorbance at 260 nm. The reverse transcription of RNA samples was performed using the iScript cDNA synthesis kit (Bio-Rad, Segrate, Italy), according to the manufacturer’s instructions.

To quantify the expression of CLDN3, CLDN5, OCLN, TJP1, IL-6, IL-10, IL-22 and TNF-α, qRT-PCR was carried out using as gene reference the ribosomal protein unit S14 coding gene. Briefly, 5 µl of iTaq Universal SYBR Green Supermix (Bio-Rad Laboratories), 2 µl of 5 µM primers mix (Table 5) and 3 µl of cDNA (5 ng/µl) were used for each sample. Samples were run using a CFX96 Real-Time System (Bio-Rad Laboratories) for 30 s at 95 °C, 5 s at 95 °C and 30 s at 60 °C for 42 cycles. The analysis was performed using Bio-Rad CFX Maestro software (Bio-Rad Laboratories).

**Table 5:** Sequences of qRT-PCR primers

Gene	Forward (5′–3′)	Reverse (5′–3′)
TJP1	CCCCACTCTGAAAATGAGGA	ACAGCAATGGAGGAAACAGC
OCLN	ATGCCATGGGACTGTCAACT	TTTGTGGGACAAGGAACACA
CLDN3	CCTGCGTCTGTCCCTTAGAC	CACGCGAGAAGAAGTACACG
CLDN5	GCTGTTTCCATAGGCAGAGC	CCCTGCCGATGGAGTAAAGA
TNF	TGGGATCATTGCCCTGTGAG	GGTGTCTGAAGGAGGGGGTA
IL6	GGTACATCCTCGACGGCATCT	GTGCCTCTTTGCTGCTTTCAC
IL10	AGACAGACTTGCAAAAGAAGGC	TCGAAGCATGTTAGGCAGGTT
IL22	GCTGCCTCCTTCTCTTGG	GTGCGGTTGGTGATATAGG
S14	AGGTGCAAGGAGCTGGGTT	TCCAGGGGTCTTGGTCCTATTT

### Statistical analysis

Statistics were performed using ANOVA (ANalysis Of VAriance) with post-hoc Tukey Honestly Significant Difference Test Calculator for comparing multiple treatments [137], using Statistical Package for Social Science software (IBM SPSS Statistics v.19). *p* < 0.05 was considered significant.

## Supplementary Information

**Additional file 1. Figure S1**. Optical appearance of the colloidal suspensions before A) and after B) treatment with the SHDS.** Figure S2**. Size distribution of the samples directly incubated in SIF or after complete SHDS-treatment of A) LSNPs; B) CNPs, C) FNPs; D) HNPs.** Figure S3**. HNPs dissolution.** Figure S4**. Degradation of LSNPs by lipase. Table S1. Mean dH and PDI of NMs in DMEM+10% FBS (100 mg/ml), 24 h incubation.** Figure S5**. Aspect of the suspension of the (*) untreated and (**) SHDS-treated A) LSNPs, B) CNPs, C) FNPs and D) HNPs in cell medium.** Figure S6**. SDS-PAGE analysis showing the hard corona of (*) SHDS-treated and (**) untreated A) LSNPs, B) CNPs, C) FNPs and D) HNPs in cell medium and obtained by centrifugation, and E) ctrl without NBMs.** Figure S7**. LFQ intensity for different proteases inhibitor for FNPs untreated and SHDS-treated.** Figure S8**. Cells viability of A) Caco-2 cells and B) HCoEpiC cells after 24 h of incubation with SHDS fluids.** Figure S9**. Genotoxicity of HCT116 cells after 24 h of incubation with untreated and SHDS-treated NMs.** Table S2**. Tran-Epithelial Electrical Resistance (TEER) values of Caco-2 barrier model after 24 h of incubation.** Figure S10**. Scheme of representative SDHS treatment.

## Data Availability

All data generated or analysed during this study are included in this published article and its additional files.

## Abbreviations

53BP1: Tumour Suppressor P53-binding Protein 1
ANOVA: ANalysis Of VAriance
BSA: Bovine serum albumin
CLDN: Claudin encoding gene
CNPs: Carbon nanoparticles
d_H_: Hydrodynamic diameter
DLS: Dynamic light scattering
DMEM: Dulbecco's modified eagle medium
ELS: Electrophoretic light scattering
EPR: Electron paramagnetic resonance spectroscopy
FBS: Foetal bovine serum
FNPs: Magnetite nanoparticles
FPIA: Flow particle imaging analysis
GI: Gastro-intestinal
HBSS: Hanks’ Balanced Salt Solution
HNPs: Hydroxyapatite nanoparticles
Ig: Immunoglobulin
IL: Interleukin
LFQ: Label free quantification
LSNPs: Lipid surfactant nanoparticles
NBM: Nano-biomaterial
NTA: Nanoparticle tracking analysis
OCLN: Occludin encoding gene
OGI: Oral-gastro-intestinal
Papp: Apparent permeability
PBS: Phosphate buffered saline
PDI: Polydispersity index
PEG: Poly(Ethylene glycol)
PLGA: Poly(lactic-co-glycolic acid)
qRT-PCR: Quantitative Real Time-Polymerase Chain Reaction
RFU: Relative fluorescence units
SBF: Simulated bile fluid
SD: Standard deviation
SDF: Simulated duodenal fluid
SDS-PAGE: Sodium Dodecyl Sulphate-PolyAcrylamide Gel Electrophoresis
SEM: Standard error of the mean
SGF: Simulated gastric fluid
SHDS: Simulated human digestion system
SIF: Simulated intestinal fluid
SSF: Simulated saliva fluid
TEM: Transmission electron microscope
TJs: Tight junctions
TNF: Tumour necrosis factor
TJP: Zonula Occludens encoding gene
