# Supplementary material for: Changes of physico-chemical properties of nano-biomaterials by digestion fluids affect the physiological properties of epithelial intestinal cells and barrier models
Source: Part Fibre Toxicol. 2022 Jul 19;19:49. doi: 10.1186/s12989-022-00491-w (PMC9297619; doi:10.1186/s12989-022-00491-w)
Supplement: Supplementary file 1 — Additional file 1. Figure S1. Optical appearance of the colloidal suspensions before A) and after B) treatment with the SHDS. Figure S2. Size distribution of the samples directly incubated in SIF or after complete SHDS-treatment of A) LSNPs; B) CNPs, C) FNPs; D) HNPs. Figure S3. HNPs dissolution. Figure S4. Degradation of LSNPs by lipase. Table S1. Mean dH and PDI of NMs in DMEM+10% FBS (100 mg/ml), 24 h incubation. Figure S5. Aspect of the suspension of the (*) untreated and (**) SHDS-treated A) LSNPs, B) CNPs, C) FNPs and D) HNPs in cell medium. Figure S6. SDS-PAGE analysis showing the hard corona of (*) SHDS-treated and (**) untreated A) LSNPs, B) CNPs, C) FNPs and D) HNPs in cell medium and obtained by centrifugation, and E) ctrl without NBMs. Figure S7. LFQ intensity for different proteases inhibitor for FNPs untreated and SHDS-treated. Figure S8. Cells viability of A) Caco-2 cells and B) HCoEpiC cells after 24 h of incubation with SHDS fluids. Figure S9. Genotoxicity of HCT116 cells after 24 h of incubation with untreated and SHDS-treated NMs. Table S2. Tran-Epithelial Electrical Resistance (TEER) values of Caco-2 barrier model after 24 h of incubation. Figure S10. Scheme of representative SDHS treatment. [file 12989_2022_491_MOESM1_ESM.docx]

**Changes of physico-chemical properties of nano-biomaterials by digestion fluids affect the physiological properties of epithelial intestinal cells and barrier models.**

Giulia Antonello^1,2,3^, Arianna Marucco^4^, Elena Gazzano^4^, Panagiotis Kainourgios^5^, Costanza Ravagli^6^, Ana Gonzalez-Paredes^7^_,_ Simone Sprio^8^, Esperanza Padín-González^9^, Mahmoud G. Soliman^9^, David Beal^10^, Francesco Barbero^1^, Paolo Gasco^7^, Giovanni Baldi^6^, Marie Carriere^10^, Marco P. Monopoli^9^, Costas A. Charitidis^5^, Enrico Bergamaschi^2^, Ivana Fenoglio^1*§^, Chiara Riganti^3*§^

*^1^ Department of Chemistry, University of Turin, Via Pietro Giuria 7, 10125 Torino, Italy.*

*^2^ Department of Public Health and Pediatrics, Piazza Polonia, 94, University of Turin, 10126 Torino, Italy.*

*^3^ Department of Oncology, University of Turin, Via Santena 5 bis, 10126 Torino, Italy.*

*^4^ Department of Life Sciences and Systems Biology, University of Turin, Via Accademia Albertina 13, 10123 Torino, Italy.*

*^5^ Research Unit of Advanced, Composite, Nano-materials and Nanotechnology, School of Chemical Engineering, National Technical University of Athens, 9 Heroon Polytechniou St., Zographos, 15780 Athens, Greece.*

*^6^ Colorobbia Consulting srl, Headwork, Via Pietramarina, 53, 50059 Sovigliana - Vinci, Firenze, Italy.*

*^7^ Nanovector Srl, Headwork, Via Livorno 60, 10144 Torino, Italy.*

*^8^ National Research Council - Institute of Science and Technology for Ceramics ISTEC-CNR, Via Granarolo 64, I48018 Faenza (RA), Italy.*

*^9^ Department of Chemistry, Royal College of Surgeons in Ireland (RCSI), 123 St Stephen Green, Dublin 2, Ireland.*

*^10^ Université Grenoble Alpes, CEA, CNRS, IRIG, SyMMES-CIBEST, F-38000 Grenoble, France.*

*Corresponding authors.

^§^Equal contribution.

E-mail addresses: [ivana.fenoglio@unito.it](mailto:ivana.fenoglio@unito.it) (I. Fenoglio), [chiara.riganti@unito.it](mailto:chiara.riganti@unito.it) (C.Riganti).

*Supplementary Information*

*
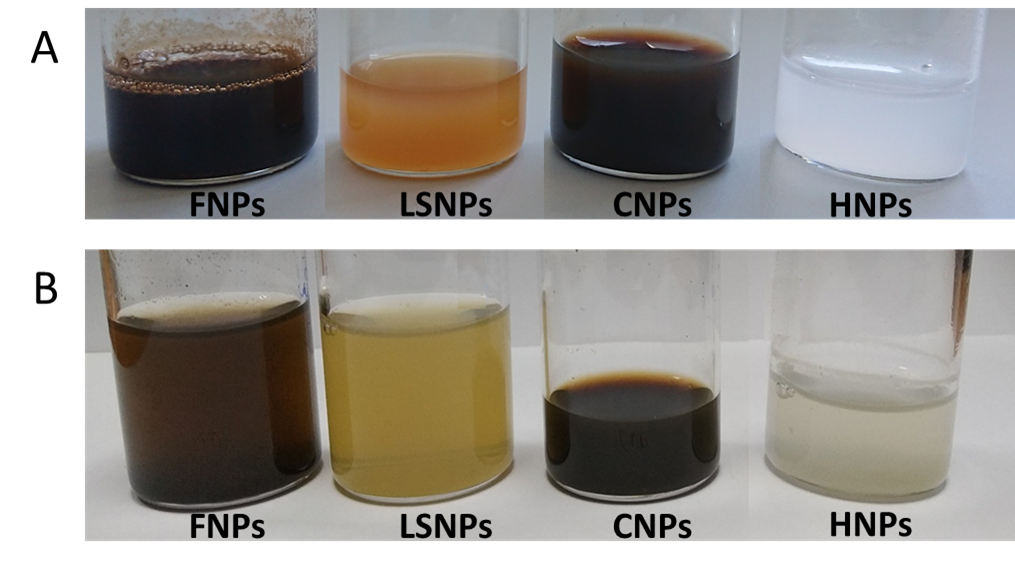
*

**Figure S1.** Optical appearance of the colloidal suspensions before A) and after B) treatment with the SHDS.


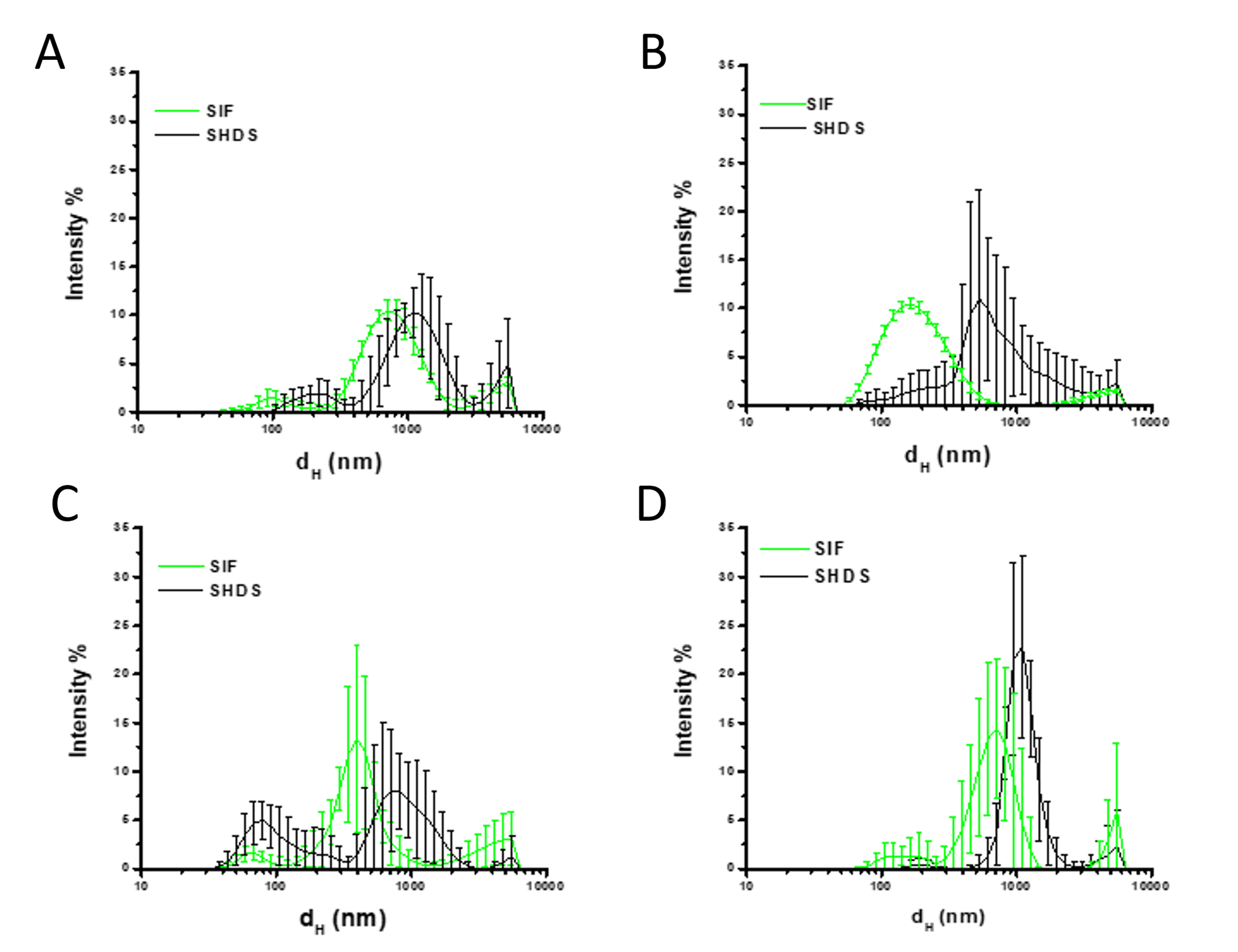


**Figure S2**. Size distribution of the samples directly incubated in SIF or after complete SHDS-treatment of A) LSNPs; B) CNPs, C) FNPs; D) HNPs.


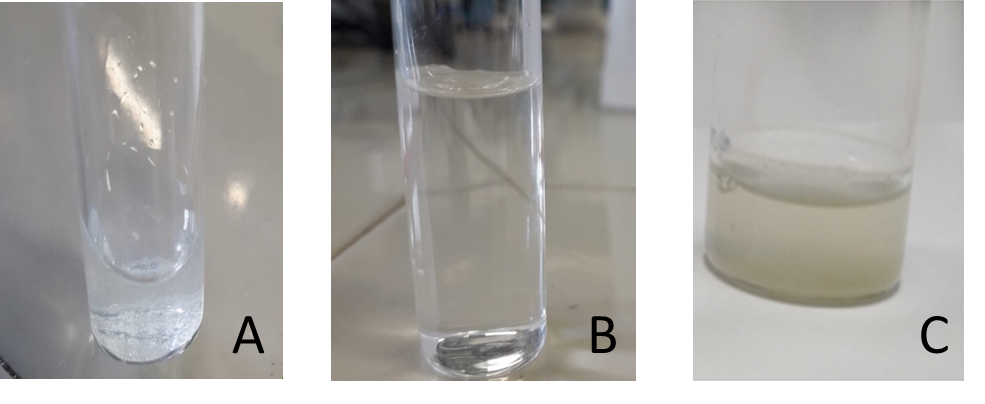


**Figure S3.** HNPs dissolution. A) HNPs suspension in water and B) in water at pH 1.4 (gastric pH) for 30 min; C) HNPs suspension at the end of treatment with SHDS.


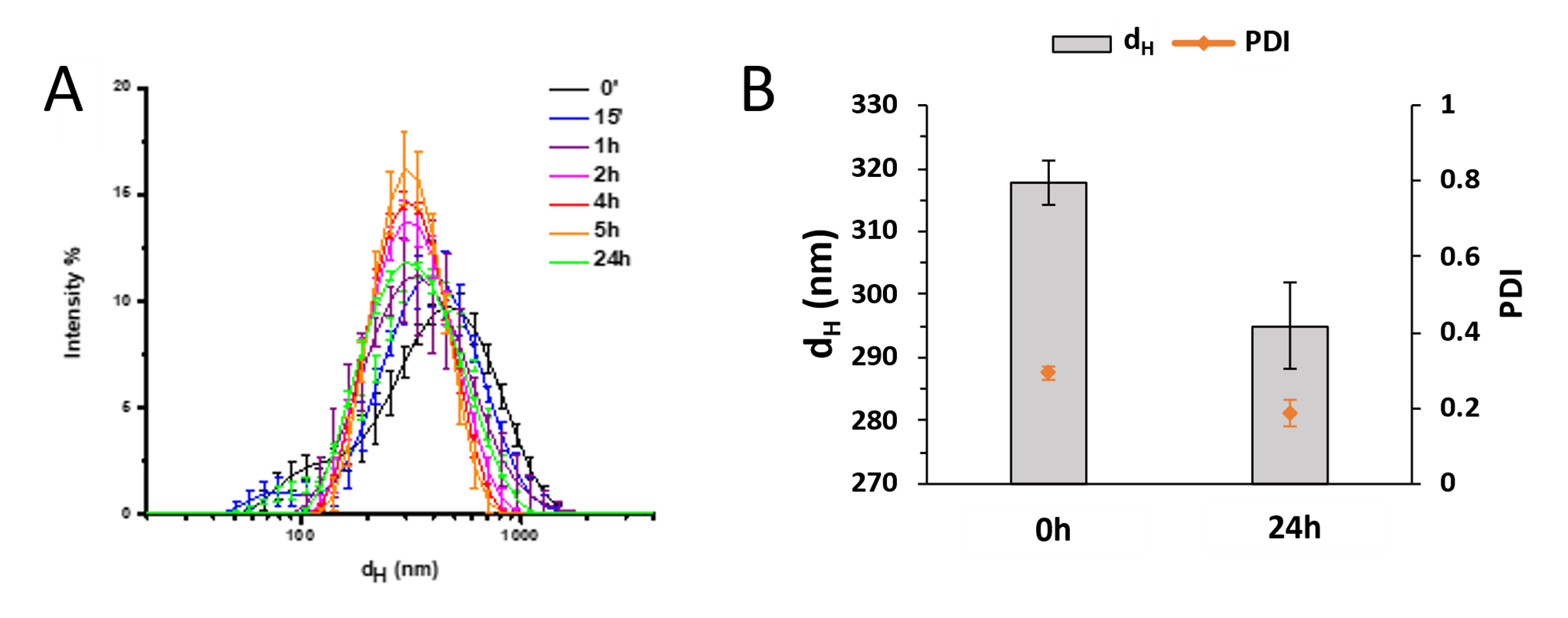


**Figure S4.** Degradation of LSNPs by lipase. A) size distribution changes occurring during the incubation of LSNPs with 0.5 mg/ml lipase (DLS); B) mean d_H_ and PDI before and after the treatment.

**Table S1.** Mean d_H_ and PDI of NMs in DMEM+10% FBS (100 mg/ml), 24 h incubation

|  | **Z-average (nm)** | **PDI** |
| --- | --- | --- |
| **LSNPs** | 281.2 ± 9.3 | 0.418 ± 0.025 |
| **LSNPs-SHDS** | 202.1 ± 5.4 | 0.613 ± 0,076 |
| **CNPs** | 188.8± 2.4 | 0.083±0.009 |
| **CNPs-SHDS** | 460.0± 31.5 | 0.487±0.032 |
| **FNPs** | 169.8 ± 3.3 | 0.300 ± 0.039 |
| **FNPs-SHDS** | 849.0 ± 6.6 | 0.522 ± 0.064 |
| **HNPs** | 653.2 ± 18.6 | 0.337±0.057 |
| **HNPs-SHDS** | 2435 ± 306 | 0.841±0.223 |

.


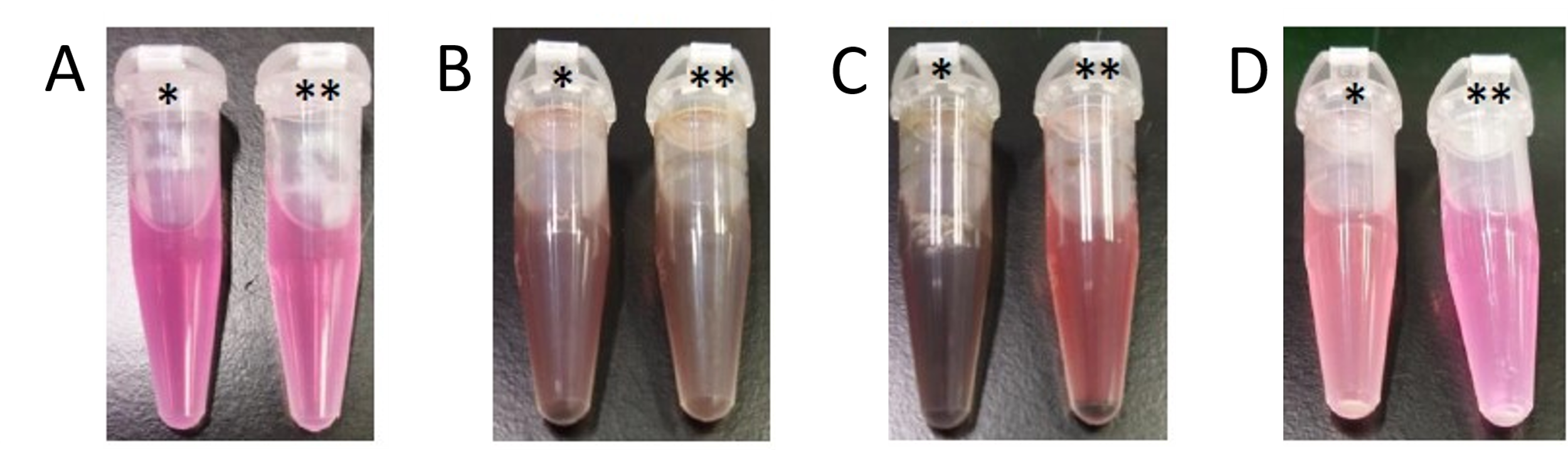


**Figure S5.** Aspect of the suspension of the (*) untreated and (**) SHDS-treated A) LSNPs, B) CNPs, C) FNPs and D) HNPs in cell medium.


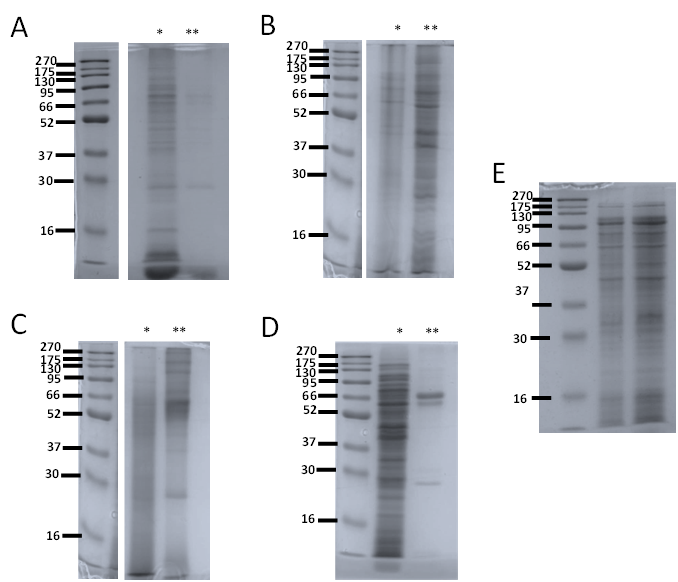


**Figure S6**. SDS-PAGE analysis showing the hard corona of (*) SHDS-treated and (**) untreated A) LSNPs, B) CNPs, C) FNPs and D) HNPs in cell medium and obtained by centrifugation, and E) ctrl without NBMs.


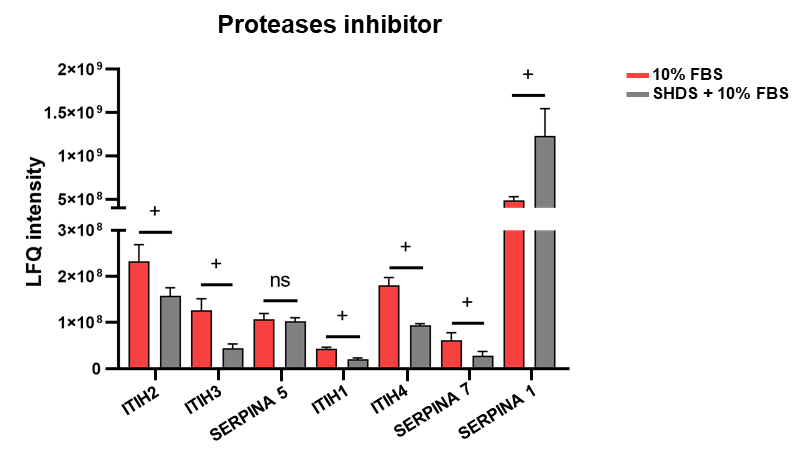


**Figure S7**. LFQ intensity for different proteases inhibitor for FNPs untreated and SHDS-treated. n=3; +p<0.05. ITIH2**:** Inter-alpha-trypsin inhibitor heavy chain H2; ITIH3: Inter-alpha-trypsin inhibitor heavy chain H3; SERPINA 5: Plasma serine protease inhibitor; ITIH1: Inter-alpha-trypsin inhibitor heavy chain H1; ITHI4: Inter-alpha-trypsin inhibitor heavy chain H4; SERPINA 7: Serpin peptidase inhibitor, clade A (Alpha-1 antiproteinase, antitrypsin), member 7; SERPINA 1: Alpha-1-antiproteinase.


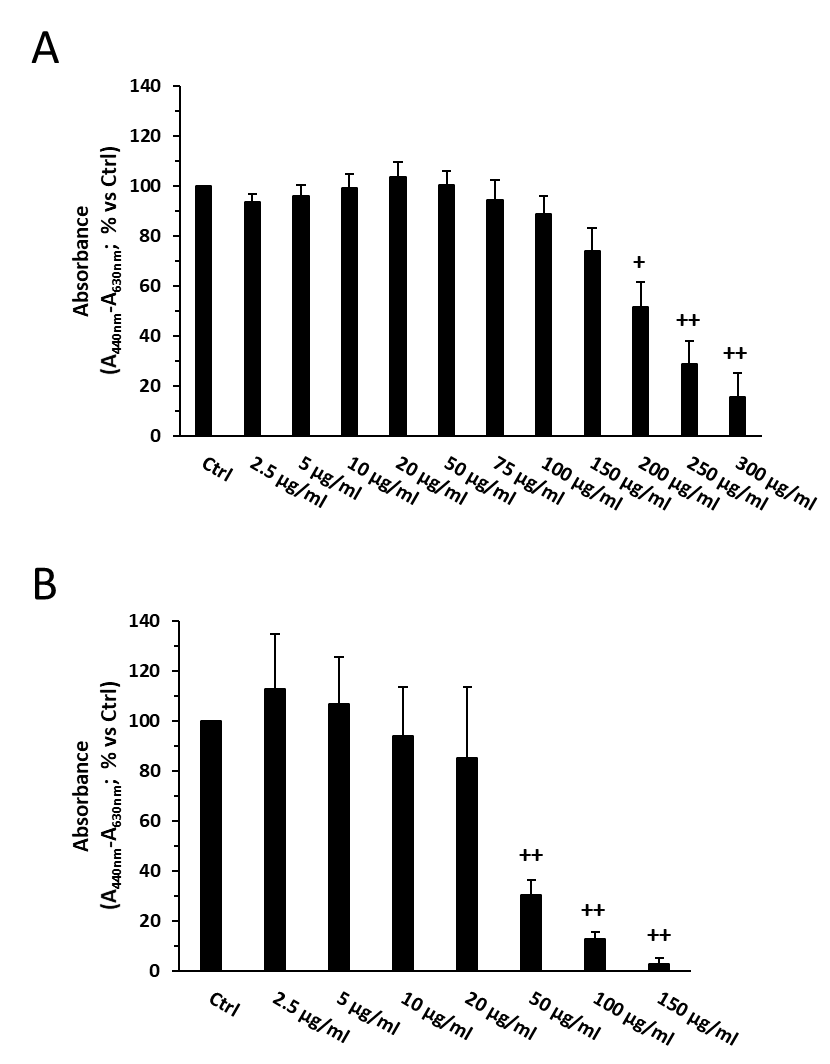


**Figure S8.** Cells viability of A) Caco-2 cells and B) HCoEpiC cells after 24 h of incubation with SHDS fluids. n=3; +p<0.05 *vs* Ctrl; ++p<0.01 *vs* Ctrl


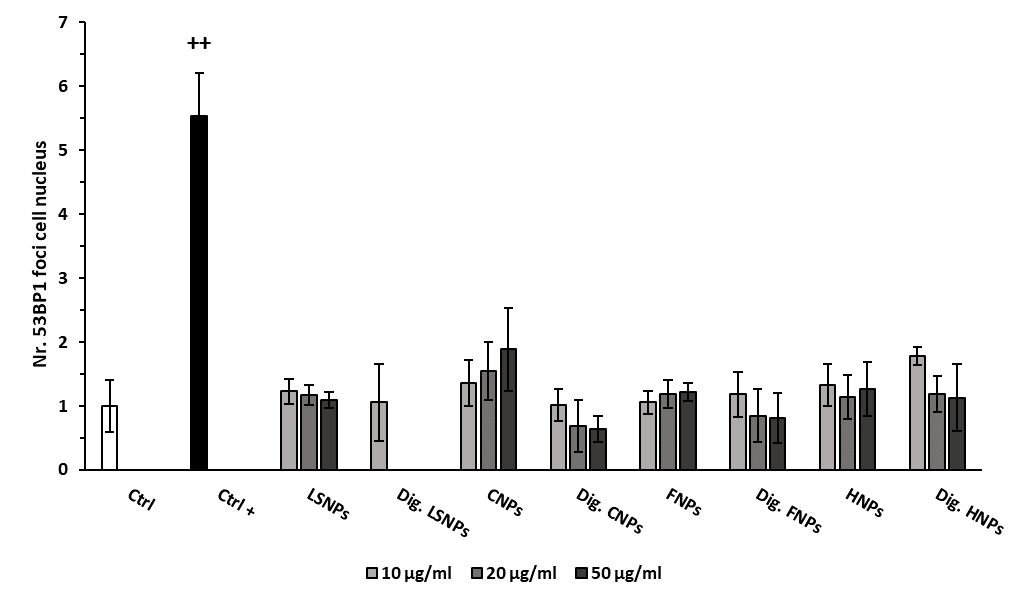


**Figure S9*.*** Genotoxicity of HCT116 cells after 24 h of incubation with untreated and SHDS-treated NMs.

+p<0.05 *vs* Ctrl; ++p<0.01 *vs* Ctrl

**Table S2**. Tran-Epithelial Electrical Resistance (TEER) values of Caco-2 barrier model after 24 h of incubation.

|  | **Untreated TEER (Ω*cm^2^)** | **SHDS-treated TEER (Ω*cm^2^)** |
| --- | --- | --- |
| **Ctrl** | 619.5 ± 44.2 | 657.3 ± 25.3 |
| **LSNPs** | 642.3 ± 31.7 | 661.5 ± 21.2 |
| **CNPs** | 674.1 ± 53.4 | 632 ± 23.1 |
| **FNPs** | 665.7 ± 37.8 | 653.2 ± 24.9 |
| **HNPs** | 623.7 ± 19.2 | 642.5 ± 23.6 |

24h of incubation with 50 µg/ml of LSNPs and FNPs, and 150 µg/ml of CNPs and HNPs. n=3; mean+/-SEM.


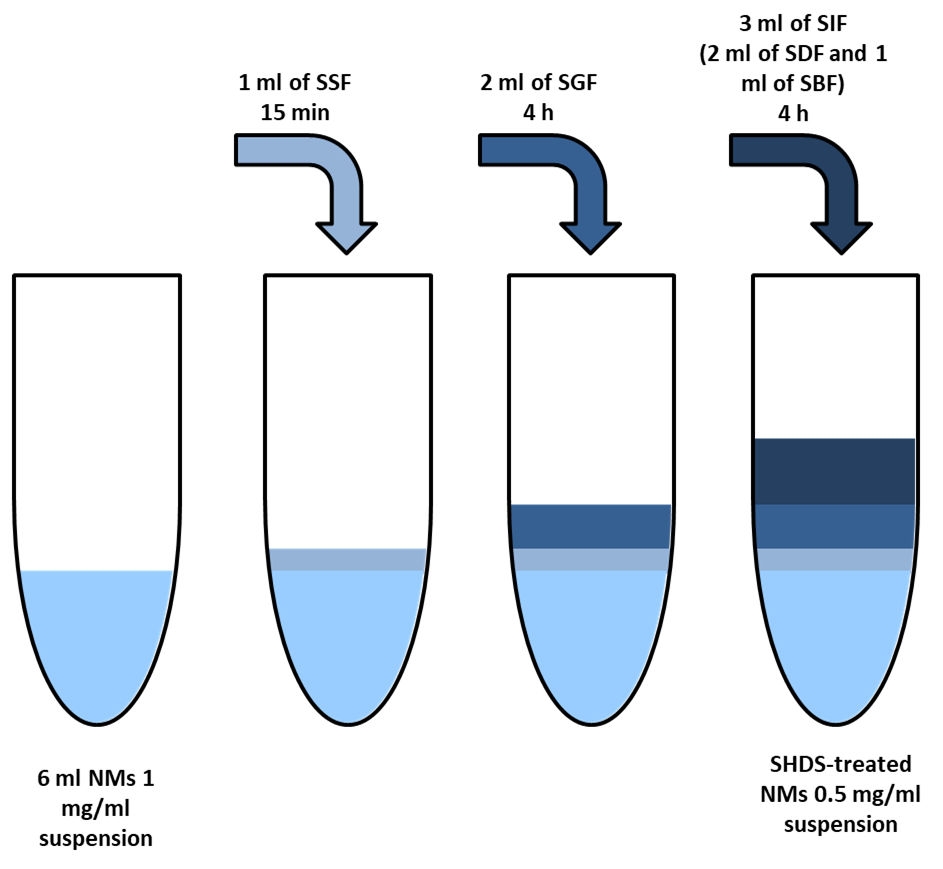


**Figure S10.** Scheme of representative SDHS treatment.
